# Protocols for isolation and characterization of nanoparticle biomolecular corona complexes

**DOI:** 10.3389/ftox.2024.1393330

**Published:** 2024-07-23

**Authors:** Mahmoud G. Soliman, Alberto Martinez-Serra, Marko Dobricic, Duong N. Trinh, Jack Cheeseman, Daniel I. R. Spencer, Marco P. Monopoli

**Affiliations:** ^1^ Chemistry Department, RCSI (Royal College of Surgeons in Ireland), Dublin, Ireland; ^2^ Physics Department, Faculty of Science, Al-Azhar University, Nasr City, Egypt; ^3^ Ludger Ltd., Culham Science Centre, Abingdon, United Kingdom

**Keywords:** nanoparticles, nanosafety, biomolecular corona, isolation methods, physico-chemical characterization, glycan analysis, proteomics

## Abstract

Engineered nanoparticles (NPs) pose a broad spectrum of interesting properties that make them useful for many applications. However, continuous exposure to NPs requires the need to deeply understand the outcomes when these NPs interact with different biological environments. After exposure within (to) these environments, the pristine surfaces of NPs strongly interact with the molecules from the surrounding medium, including metabolites, lipids, glycan, and proteins, forming the so-called protein corona (PC). It is well established that the NP-PC strongly influences the biological fate of various NPs types, including cellular uptake, toxicity, and biodistribution. Thus, for a proper assessment of potential hazards associated with engineered NPs, it is mandatory to study and evaluate the PC that forms around NPs. Herein, we describe protocols in detail for the isolation and characterization of NP-PC complexes and cover the following aspects: 1) isolation protocols for different nanomaterials in a range of exposing media, including magnetic isolation methods for superparamagnetic NPs, 2) NP physico-chemical characterization using advanced and standard techniques available in regular laboratories, and 3) NP- PC characterization of the protein and glycan components.

## 1 Introduction

Engineered nanoparticles (NPs) of nanometric size are of great interest because of their unique physio-chemical properties, including their particle size, shape, composition, surface area, surface reactivity, and surface charge. (Whitesides, 2005; Wolfram et al., 2015; Zhang et al., 2016). Rapid progress in nanotechnology led to the development of various nano-based products for many applications, including food, cosmetics, microelectronic devices, pharmaceuticals, etc. (Ravichandran, 2010; Raj et al., 2012; Marchesan and Prato, 2013; Contado, 2015) Currently, several thousand types of nanomaterials are available in the market, (Podsiadlo et al., 2007; Mehta et al., 2012; Aslani et al., 2014), and it is expected to increase over the next years. As the use of NPs continues to grow exponentially, with advanced and multicomponent nanomaterials (MCNM) used industrially in many fields, human exposure becomes a matter of concern, which can lead to a potential risk to human health and requires a proper assessment and characterization (Nowack et al., 2012).

Because of NPs’ small size, they can enter the human body through different exposure routes, including inhalation, ingestion, injection and skin contact. For instance, NPs can enter the body through the digestive system by being ingested with food or water. They may be present in certain food products, food additives, or as contaminants. The gastrointestinal tract provides another potential route for the absorption of NPs into the blood circulation (Seaton et al., 2009). Inhaled NPs can accumulate in the alveoli of humans and animals (Halimu et al., 2022) or translocate into the circulation, leading to different levels of toxicity (Miller et al., 2017). Similarly, toxic effects were correlated with inhalation exposure to ferric oxide (Wang et al., 2010) or the long-term in vivo biotransformation of magnetic NPs (Levy et al., 2011). Other examples include the release of NPs from medically implanted devices such as leaching of NPs from hip implants or dental composites, which may have a long-term toxic impact on patients (Goode et al., 2012). In this context, it is difficult to interpret results from toxicological studies to understand the pathogenic mechanisms associated with the NPs without proper characterization of NPs and analysis of their interactions at the biological interface. In particular, the NPs establish a new series of dynamic interactions between their surfaces and biomolecules from the surrounding environment, which lead to the formation of what is called protein corona (PC) (Cedervall et al., 2007). The PC is often divided by researchers into i) hard corona (HC), where the layer directly coats the particle surface and contains proteins with a high affinity to the particle surface, which can evolve over a longer period, and ii) Soft corona (SC), where the proteins are exchangeable with the surrounding molecules because of their low affinity to the particle surface, but high abundance in the biological fluid. (Cedervall et al., 2007; Lundqvist et al., 2008; Casals et al., 2010; Lima et al., 2020).

The PC term was first introduced by Linse and Dawson in 2007, but it is now widely studied as relevant to nanomedicine, nanotoxicity and environmental science. (Cedervall et al., 2007; Nel et al., 2009; Walkey and Chan, 2012; Walkey et al., 2014; Caracciolo et al., 2017; Wheeler et al., 2021; Mahmoudi et al., 2023). Binding proteins to the NPs surface can alter their structures, which leads to the loss of their enzymatic activity, disturbance of biological processes, and acceleration the pathogenic events such as amyloidosis. On the other hand, the adsorption of proteins on the NPs surface can alter their colloidal characteristics, including NPs aggregation characteristics, and/or hydrodynamic diameter, which may affect the cellular response to the NP’s exposure, including accumulation, toxicity and clearance. (Dawson and Yan, 2021). Indeed, the challenges in understanding biomolecular corona complexes are multifaceted and require a comprehensive, interdisciplinary approach. Establishing a highly collaborative infrastructure would facilitate thorough physico-chemical characterization of materials, the identification of biomolecular components such as proteins, lipids, glycans, or DNA, and the elucidation of their biological functions. (Mahmoudi, 2022). For example, recent studies have shown that the corona can adsorb circulating DNA (Griffith et al., 2020), which significantly influences the NP-mediated immune response (Anees et al., 2024), while others have reported the connection between the glyco-corona and its effects on the *in vivo* localization of NPs (Wan et al., 2015). However, there is still a need to improve the methodologies for preparing the corona formed on NPs to ensure reproducibility and robustness. (Mahmoudi, 2022). For instance, current experimental protocols require the NP exposure to be performed under shaking conditions, which often falls short in replicating the shear forces encountered by NPs in the bloodstream, impacting their behavior and interaction with biomolecules. The knowledge on nano-bio interactions together with clear methods and protocols are crucial to obtain before transferring the NPs to industrial bodies, ensuring that only safe NPs are introduced to the market. (Petersen et al., 2022; Fadeel and Keller, 2024).

In this context, the literature is abundant with a large number of publications, which focus typically on the dynamic and composition of PC formation and the impact of PC on biomedical applications of different NPs, including distribution, toxicity, and clearance. (Monopoli et al., 2011; Lesniak et al., 2012; Gunawan et al., 2014; Bai et al., 2021). Several reviews focused on the most relevant techniques in the characterization of NPs in physiological conditions since the biological conditions can affect the NPs properties. (Hall et al., 2007; Sapsford et al., 2011; Krug, 2014; Ping-Chang Lin et al., 2014). Few other reviews focus on the interactions of NPs with biological components, methods for the isolation of NP-PC complexes, (Faserl et al., 2019; Bonvin et al., 2017; Böhmert et al., 2020; Monopoli et al., 2013a), and protocols for the preparation of proteomics samples. (Monopoli et al., 2013a; Docter et al., 2014; Faserl et al., 2019; Kruszewska et al., 2021). However, the available information is widely scattered in the current literature, and no single review article has provided a collective protocol that steadily describes *in vitro* preparation, isolation, characterization, proteomics analysis, and glyco-profiling of the PC. Therefore, this work aims to provide a solid reference protocol that highlights standard procedures to help the researchers correctly perform the experimental steps of a PC study for NPs. We provide all the steps from blood protein samples (aliquots, storage, and process), typically used for mimicking *in vitro* and *in vivo* conditions, to corona preparation, different isolation methods, characterization techniques, and sample preparation for proteomics and glyco-profiling analysis.

## 2 General considerations and recommendations

The isolation and characterization protocols described in this article can be successfully applied to all NPs, considering optimization for ultra-small NPs, particles with lower dimensions than Bohr excitation radius (Quantum Dots) and/or low-density particles (like solid-lipid NPs). These methods describe how to obtain NP-HC complexes, and despite they can be optimized to study the SC, more accurate approaches have been recently developed such as cryoTEM with synchrotron-radiation circular dichroism (CD) or *in situ* click-chemistry. (Mohammad-Beigi et al., 2020; Sanchez-Guzman et al., 2020; Bai et al., 2021).

To harmonize the *in vitro* and *in vivo* exposure of NPs, a suitable *in vitro* experiment should simulate the exposure route of NPs. This can be obtained by diluting the solution of the NPs in complex biological media that mimic the NPs exposure pathway. For instance, soaking the NPs in cell culture medium containing a specific concentration of FBS is the most common way to mimic the *in vitro* exposure studies, including cellular uptake and cytotoxicity. Inhalation exposure is usually mimicked by mixing the NPs in synthetic lung fluid (SLF) that incorporates the major components of the fluid that lines the human lung. Exposure of the NPs to the gastrointestinal (GI) tract upon oral exposure involves sequential incubations of the NPs in the environments that simulate the environments in the mouth, stomach, and duodenum of the small intestine. In this context, three commonly used simulated fluids are simulated saliva fluid (SSF), simulated gastric fluid (SGF), and simulated intestinal fluid (SIF), which contain the components that replicate the conditions of the human digestion system. In contrast, for investigating systemic circulation, the addition of blood proteins is prerequisite for a reliable cell exposure to NPs. It is important to note that the composition of biological fluid may vary depending on the specific research objectives and the requirements of the experimental setup. Researchers may adjust the formulation of biological fluid to better mimic physiological conditions or to suit the needs of their particular study. The next step of PC preparation requests an optimization of NPs incubation conditions with the biological fluids, including protein concentration, NPs concentration, incubation temperature, and fluid pH, as well as, static or dynamic exposure, for mimicking those conditions present *in vivo*.

## 3 Material and reagents

Human blood plasma (plasma) or serum can be obtained from multiple sources, such as commercial sources, local blood transfusion centers, blood transfusion from healthy volunteers or a cohort of disease patient groups. IONPs were received from Colorobbia under the funding of the European Union’s Horizon 2020 research and innovation program (BioRima project, grant agreement No 760928). Gold NPs and gold nanorods were synthesized according to established protocol by Soliman et al. (Soliman et al., 2015) Imperial™. Silica NPs (PSI-0.1) were purchased from Kisker Biotech (Germany). Carboxylated polystyrene microspheres 0.10 μm (16,688) were purchased from Polysciences. Tris-HCl, glycerol, sodium dodecyl sulfate (SDS), ammonium bicarbonate, formic acid (FA), ethylenediaminetetra acetic acid (EDTA), dithiothreitol (DTT), phosphate buffer saline (PBS), acetonitrile (ACN), agarose, iron atomic spectroscopy standard solution, acetic acid, hydrochloric acid, orange G, hydrogen peroxide (H2O2), Iodoacetamide, Sucrose, uranyl acetate, Tris-borate, Tris-acetate, indole-3-acetic acid, ammonium bicarbonate, trifluoroacetic acid, dimethyl sulfoxide (DMSO), and β-mercaptoethanol were purchased from Merck Life Science Limited (Ireland). Protein Stain 40% acrylamide/bisacrylamide, and SDS-PAGE Loading Buffer were purchased from Thermo Fischer Scientific Ireland. Orange G loading dye was purchased from Alfa Aesar. Neodymium external magnetic bar (42 x 8 × 10 mm thick N42 Neodymium Magnet - 14 kg Pull) was purchased from first4magnets (United Kingdom). The LudgerZyme PNGaseF (LZ-PNGF-150), Ludger Tag Procainamide Glycan Labeling Kit (LT-KPROC-24), Ludger-Clean Procainamide Clean-up Plate (LC-PROC-96), 50 mM ammonium formate buffer pH 4.4 LS-N-BUFFX40), LudgerTag™ DMB Sialic Acid (LT-KDMB-96) and LudgerSep-uR2 UHPLC column were purchased from Ludger Ltd. (United Kingdom). The ACQUITY UHPLC BEH-Glycan 1.7 μm, 2.1 × 150 mm column was purchased from Waters. Carbon film 300 mesh TEM grids were purchased from Agar Scientific (United Kingdom). Superdex 75 Prep Grade was purchased from Cytiva (Ireland). PVC Standard 0.544 nm was purchased from Analytik Ltd. (United Kingdom). Eppendorf microcentrifuge tubes were used for all experiments. The use of low protein binding tubes is recommended to reduce plasma protein and NPs adsorption. All solutions and suspensions were prepared in double-distilled water (resistivity of 18.2 MΩ cm). Protective equipment must be always worn (safety glasses, disposable gloves, and a laboratory coat).

## 4 Methods

### 4.1 Blood plasma biobank preparation

Typically, PC studies rely on the use of a biobank of healthy donors. Blood plasma or serum can be obtained from local blood transfusion centers or following established procedures (Rai et al., 2005). In order to create a biobank, mix an equal amount of plasma of at least six donors and aliquot and store at −80°C, avoiding freeze/thaw cycles for sample preservation. Leftover biological fluid must be discarded on the day of the experiment.

### 4.2 NPs incubation with biological fluid

1. Allow the biological fluid under investigation, e.g., plasma or serum, to reach room temperature or the incubation temperature (37°C). Plasma is typically diluted in PBS containing EDTA to reach a final concentration of 1 mM EDTA to avoid possible blood plasma activation. (Monopoli et al., 2013a).
2. Vortex the whole fluid to ensure homogeneity of the solution.
3. Centrifuge at 16,000 rcf for 3 min to remove possible aggregates present in the solution. Then, transfer the supernatant to another microcentrifuge tube, and discard the pellet (aggregates).
4. Using PBS, prepare the desired dilution of proteins, for instance, 10% or 80% plasma to mimic *in vitro* or *in vivo* conditions, respectively, and then vortex briefly to ensure homogeneity.
5. Add NPs to the biological solution and mix gently using a pipette to ensure the dispersity of particles in the solution. A standard concentration of NPs used for PC study is 0.1 mg/mL. (Liu et al., 2020). However, this can vary based on the specific characteristics of the material, such as the type of particle core or particle size. (Faserl et al., 2019). Researchers are encouraged to adjust it based on the specific characteristics of their NPs and align as match as possible to the exposing scenario. Be aware that varying the ratio between surface area and biomolecules present in the media can affect the NPs’ colloidal stability and composition, therefore this item should be taken into account. (Trinh et al., 2022).
6. Place the samples in a thermal shaker at a mixing speed of 300 rpm at 37°C (a general incubation temperature for corona study) and for a period of time that matches the experiment/study requirements.

### 4.3 NPs corona preparation

#### 4.3.1 Isolation by centrifugation

**FIGURE 1:**
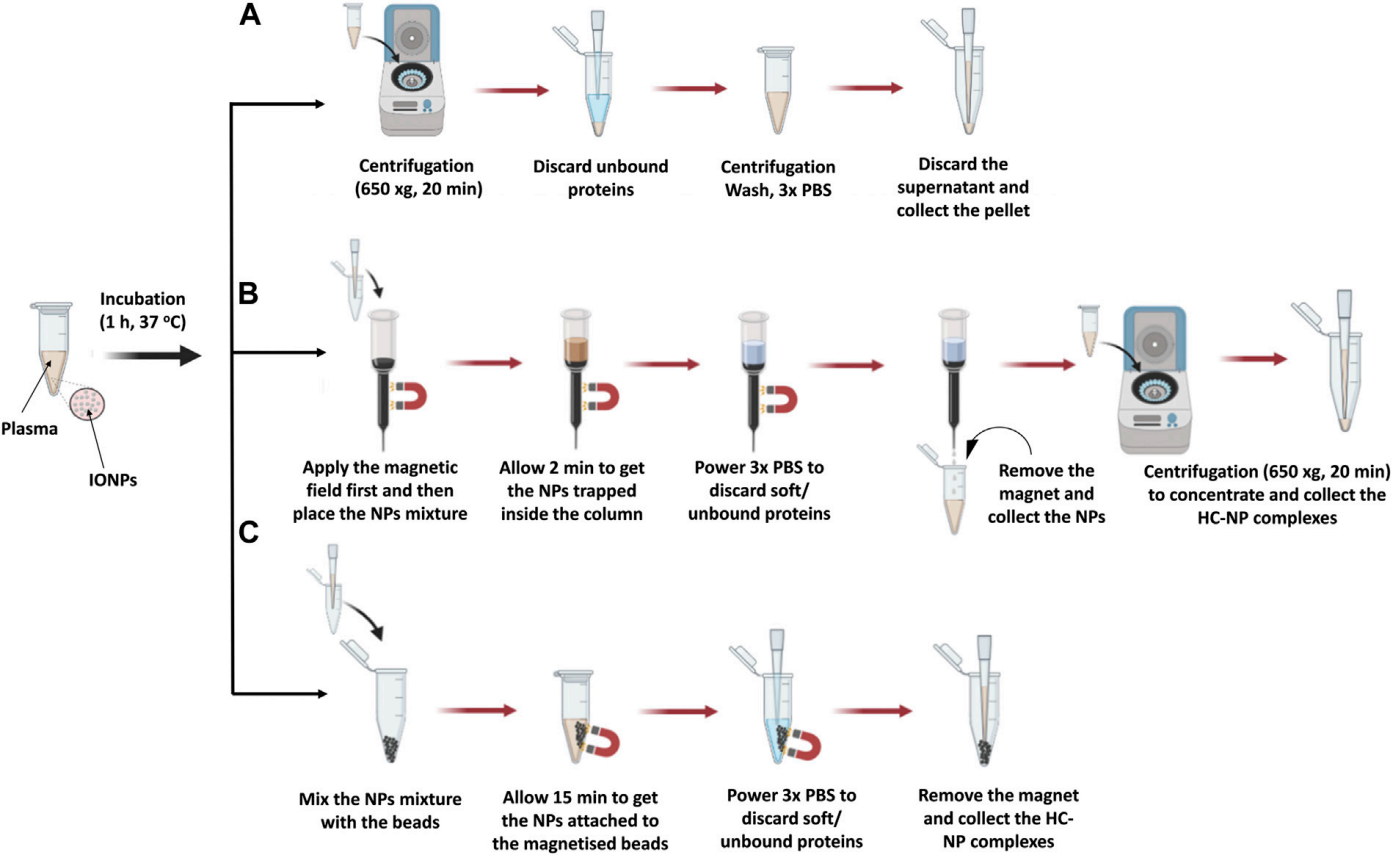
Illustration of the isolation steps of NP-HC complexes after exposure to human blood plasma using **(A)** centrifugation, **(B)** MACS® column, and **(C)** MagBed. Reprinted from the study by Soliman et al., 2024, Copyright (2024), with permission from Elsevier.

Common protocols rely on the use of benchtop centrifugation of NP corona isolation as it is commonly available in research laboratories. The centrifugation time and speed are dependent on the NP size and density. The NP-HC complexes are usually pelleted by applying a centrifugal force since they are denser than the other biomolecules present in the fluid. After centrifugation, the supernatant is discarded and the pellet is resuspended in the proper buffer, promoting the diffusion of the loosely bound proteins (SC) into the buffer while the tightly bound proteins stay attached to the surface of the NPs. This process is usually repeated three times to acquire the NP-HC complexes - after this, subsequent washing cycles are unsuccessful in removing extra proteins. Long centrifugation times are to be avoided as they may promote the pelleting of protein aggregates with the NP-HC complexes, leading to identifying the abundant protein aggregates as a portion of PC. To minimize this and remove protein aggregates, the pristine fluid should be centrifuged before use. For instance, human plasma is centrifuged at 16,000 rcf for 3 min prior to incubation. Separation by centrifugation might not be desirable for low-density NPs, such as liposomes, as they will lead to protein aggregates during the sedimentation time. A typical protocol for the separation by centrifugation is provided below and in Figure 1A.

1. Set the sedimentation speed and time of the centrifuge to match the particle size and density. This step must be pre-optimized to ensure no NPs are left in the supernatant. Avoid using a centrifugal speed and time above the required to precipitate NP-corona complexes as this can lead to particle aggregation and/or sedimentation of unbound proteins.
2. Adjust the temperature to the optimal conditions for the experiment (standard temperature for protein centrifugation set at 4°C). (Clemente et al., 2022).
3. Immediately after the desired incubation time is finished, place microcentrifuge tube of biological mixture-containing NPs in a benchtop centrifuge.
4. Centrifuge the samples until all NP-SC complexes have been pelleted.
5. Carefully remove the supernatant without disturbing the pellet, add 0.5 mL of PBS, and gently mix the solution using a pipette to ensure the particles are resuspended. Use the vortex briefly to help the resuspension of particles, if needed. Avoid using sonication as this can dissociate corona proteins from the particle surface.
6. Spin the NP-corona complexes down using the centrifugation conditions optimized previously in Step 1.
7. Gently remove the supernatant (wash 1), add 1 mL of PBS and resuspend the particle as in Step 4. It is strongly recommended to transfer the resuspended solution into a new microcentrifuge tube to avoid contamination.
8. Spin the NP-corona complexes down using the centrifugation conditions optimized previously in Step 1.
9. Gently discard the supernatant (wash 2), add 1 mL of PBS and resuspend the particle as in Step 4.
10. Spin the NP-corona complexes down using the centrifugation conditions optimized previously in Step 1.
11. Gently discard the supernatant (wash 3), add an appropriate amount of PBS (tuned to the subsequent analysis) and collect the pellet of the NP-HC complexes, which should be prepared freshly before any of the techniques listed in Subheadings 4.4 for the characterization of particle physicochemical properties. Note that for proteomics analysis by the analytical techniques listed in Subheadings 4.5, samples can be stored at ‒20°C and later processed according to the chosen analysis protocol.

#### 4.3.2 Magnetic isolation with MACS® column

MACS® Column-based cell separation is a widely used technique in research, clinical diagnostics, and cell therapy applications. It enables the rapid and gentle isolation of specific cell populations from complex mixtures by leveraging their binding to antibodies conjugated to magnetic microbeads packed inside the column. These microbeads are ferromagnetic particles, which get magnetized when the MACS® column is placed in a magnetic field. Upon removal from the magnetic field, labeled cells are smoothly released, and ready to be collected for further applications. (Miltenyi Biotec, 2024). Similarly, magnetic NPs can be isolated using the MACS® column following exposure to a complex mixture, with retention within the column achieved through external magnetic forces. Subsequent washing steps remove unbound proteins and surrounding solvent constituents. Elution of the NP occurs upon removal of the magnetic field, rendering this method suitable for rapid separation of superparamagnetic NPs from non-dense media wherein entrapment could occur. (Mahmoudi et al., 2011; Soliman et al., 2024). A detailed protocol is provided below and in Figure 1B.

1. Hold the MACS® column to a clamp.
2. Equilibrate the column by eluting 3 times the column volume (CV **≈** 1.25 mL) of the elution buffer (e.g., PBS) through it. Repeat this step two additional times to ensure full equilibration with the elution buffer.
3. Attach the magnet to the side of the column.
4. Immediately after the completion of the desired incubation time of the magnetic NPs in the biological fluid of choice, add the solution on the top the column and let the solution flow through the column. While the unbound biomolecules will elute through the column, the NPs will remain trapped into the column.
5. Add 1.25 mL of mobile phase (e.g., PBS) on top of the column four times to wash out the unbound/loosely biomolecules (SC).
6. Detach the external magnet from the column and add 1.25 mL of PBS to retrieve the NP-HC complexes from the column and collect the elution media
7. If needed, concentrate the collected solution of NP-HC complexes using a bench centrifuge by applying the sedimentation speed and time that match the NPs size and density.
8. Gently discard the supernatant, add an appropriate amount of PBS and collect the pellet of the NP-HC complexes, which should be freshly prepared before analysis. It is important to avoid freeze-thaw cycles.

#### 4.3.3 Magnetic isolation with MagBed

In this approach, ferromagnetic beads were collected from MACS® Column-based cell separation by cutting the plastic column holder and subsequently stored in a sealed plastic container until needed. These microbeads, as specified by the manufacturer, exhibit non-toxic properties and are coated with a biocompatible polymer, likely to attenuate the unspecific protein adsorption. Additionally, they can get magnetised within seconds upon exposure to an external magnetic field while maintain separation in its absence, thereby facilitating the isolation of superparamagnetic NP from complex mixtures, including those with high viscosity. Notably, all purification steps can be conducted within a microcentrifuge tube without necessitating supplementary purification steps, thereby minimizing potential sample loss. A detailed protocol is described below and in Figure 1C.

1. Weight 25 mg of microbeads in 1.5 mL microcentrifuge tube.
2. After completing the incubation of the magnetic NPs with the biological fluid, transfer the sample immediately to the microcentrifuge tube containing the microbeads.
3. Place a magnet next to the microcentrifuge tube containing the sample with the microbeads and leave it for 15 min to ensure the separation of NP-HC complexes from the surrounding biological medium.
4. Carefully remove the surrounding solution without disturbing the beads.
5. Strongly flush 1 mL of PBS inside the microcentrifuge tube using a pipette to wash out any unbound and loosely attached proteins.
6. Remove the surrounding solution slowly and without perturbing the pellet (wash 1) without touching the beads.
7. Strongly flush 3 mL of PBS into the microcentrifuge tube in three independent wash cycles to remove the unbound/loosely attached proteins.
8. Gently, remove the surrounding solution (washes 2–4) without touching the beads.
9. After the last wash cycle (wash 4), remove the external magnet and add 20 μL of PBS into the microcentrifuge tube. Pipette up and down the PBS solution for 1 min to ensure that the NP-HC complexes are retrieved from the microbeads.
10. Freshly prepare the NP-HC complexes for their characterization or store at −20°C for later analysis by techniques listed in Subheadings 4.6. It is important to avoid freeze-thaw cycles.

#### 4.3.4 Isolation by size exclusion chromatography (SEC)

**FIGURE 2:**
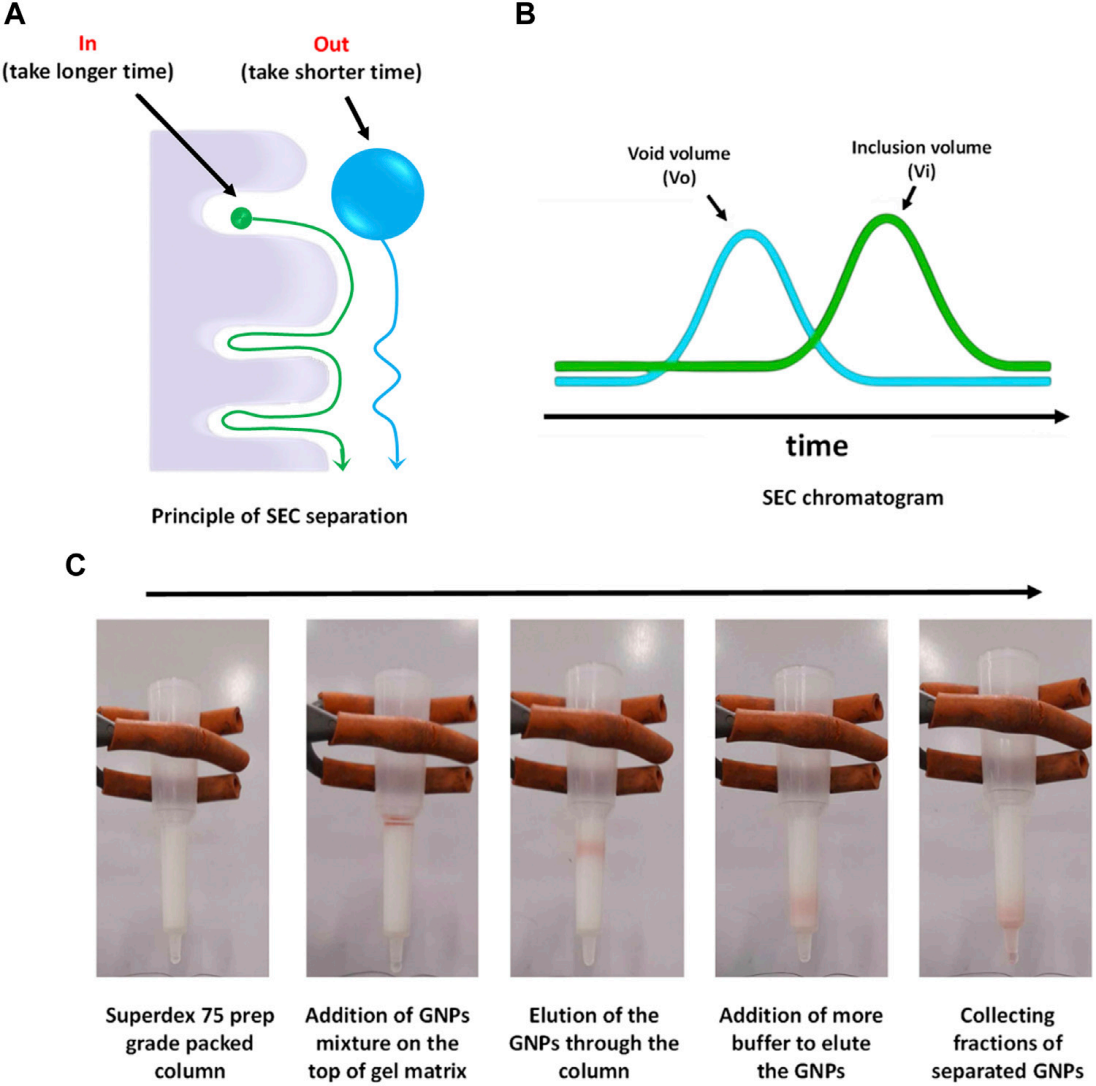
SEC conceptual scheme and procedures. **(A)** Principle of SEC separation, **(B)** typical SEC chromatogram, and **(C)** separation process of GNPs from human plasma using Superdex 75 prep grade packed in a syringe column.

Size exclusion chromatography (SEC), or gel filtration chromatography, is a technique used to fractionate molecules based on their size without altering their structure. (Brange et al., 1992; Huo, 2011; Hong et al., 2012; Hall, 2018). It has been widely used in biochemistry, molecular biology, and the pharmaceutical industry for the purification and analysis of proteins, nucleic acids, and other large biomolecules such a viruses, but it is now been used to isolate exosomes, low density NPs such as liposomes and synthetic NPs. (Hadjidemetriou et al., 2016). It is typically performed under pressure generated by a fast protein liquid chromatography (FPLC) connected to a UV detector and a fraction collector (Monopoli et al., 2013b) or by gravity. The stationary phase in SEC is packed inside a column and typically composed of pore beads with molecular dimensions and a narrow range of sizes that allow them to act like molecular sieves. The mobile phase that contains the sample solution is injected into the stationary phase. The separation process is done based on whether a protein of a particular size can enter or be excluded from the pore. Larger proteins that cannot enter the pore size will not enter into the interior volume of the beads and, therefore, will be eluted faster in what is termed the void volume (Vo) of the column. Small proteins that can enter the beads’ pores will travel a longer distance, take a longer time, and elute toward the end of the chromatogram in what is termed the inclusion volume (Vi), as shown in Figure 2 a-b. SEC gels can be made from different materials with different ranges of pore sizes that can separate a diverse range of molecular weights, including NPs, such as cross-linked agarose (Sepharose™, Bio-Gel A™), dextran (Sephadex™), cross-linked acrylamide (Sephacryl™, Bio-Gel P™), or a mixture of agarose and dextran (Superdex™). (Hong et al., 2012; Monopoli et al., 2013b; Hall, 2018). To achieve high-resolution separation, meticulous selection of column length and packed matrices resolution is essential. For instance, when the NP size is within the gel matrix resolution, they can penetrate pores, becoming resolved with the biomolecules from the fluid and not eluted with the void volume. Therefore, a larger void volume can generally enhance the yield of NPs by minimizing their entrapment. However, this may lead to the co-elution of larger macromolecules, necessitating additional steps for separation. For FPLC based SEC, it is possible to use a pre-packed column using an appropriate matrix for the NP of choice or to pack a column with a desirable size with the matrix of choice as already described (Monopoli et al., 2013b). A typical protocol for packing a syringe column using Superdex 200 prep grade by gravity is described below.

1. Gentle mix the Superdex solution.
2. Ensure the column is clean and free of any previous sample residues, in case it has been used before.
3. Pour the packing matrix/gel one shot into the column. Avoid adding the gel matrix multiple times.
4. Wait until the gel matrix settles in the syringe.
5. Don’t let the column go dry. Keep adding water to get the gel matrix fully settled. This helps to remove air bubbles and ensures even packing.
6. Equilibrate the packed column by using running the buffer through the column to equilibrate the packed beds and remove any remaining air bubbles.
7. Add the NP mixture on the top packed column and collect fractions. An example of the separation of GNPs from human plasma is shown in Figure 2C.
8. When the column is not in use, store it in an appropriate buffer to prevent drying of the packing material.

### 4.4 NPs corona physico-chemical characterization

#### 4.4.1 Dynamic light scattering

DLS is a broadly used characterization technique to measure the NP hydrodynamic size as it is simple, fast, easy to use and non-destructive to particles and biomolecules. It has a wide range of working particle sizes and concentrations that makes it suitable to characterize different types of NPs. (Murdock et al., 2008; Takahashi et al., 2008; Jans et al., 2009). DLS measures the change in the intensity of scattered lights due to the change in Brownian motion of the NP and NP-conjugates to calculate the particle diffusion coefficient, which is then related to hydrodynamic radius via the Stokes-Einstein equation. DLS provide detailed information on particle size distribution, including hydrodynamic size-based intensity distribution, intensity-weighted mean hydrodynamic size (Z-average), polydispersity index, among others (Bhattacharjee, 2016; Xu et al., 2018; Manus Maguire et al., 2018) However, DLS encounters limitations when samples exhibit high polydispersity or when NPs are embedded in complex media. In such cases, the Polydispersity Index threshold can be exceeded, leading to less reliable results. To determine if DLS is appropriate, consider the sample’s characteristics, such as size distribution and the presence of aggregates or impurities. If the sample is highly polydisperse or prone to aggregation in complex media, alternative techniques like Differential Centrifugal Sedimentation (DCS) may be more suitable, as they can provide more accurate measurements in such scenarios (section 4.4.3). A detailed protocol on the NP measurements by DLS is available at the website of the Nanotechnology Characterization Laboratory (NCL) - National Cancer Institute (Cancer, 2024) and at the website of the European Nanomedicine Characterisation Laboratory (EUNCL) (Calzolai, 2015). To achieve precise and high-quality measurements of NP-HC complexes, it is imperative to adhere to the following recommendations:

1. **Sample preparation:** Ensure thorough purification of NP-HC complexes to eliminate contaminants that could interfere with measurements. Dilute samples in an appropriate buffer (e.g., PBS) to achieve a suitable concentration range for DLS analysis, typically between 0.1 and 1 mg/mL. Ensure complete dispersion, avoiding tip sonication/any type sonication. The sample must be freshly prepared before the measurement.
2. **Data acquisition:** Acquire multiple measurements for each sample to ensure statistical reliability and consistency.

**FIGURE 3:**
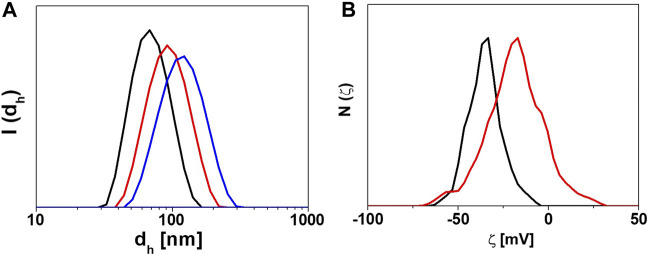
Characterization of the physicochemical properties of NP-HC complexes. **(A)** DLS intensity distribution of the hydrodynamic diameters (I_(dh)_) of gold NPs before (black) and after exposure to human plasma, 10% (red) and 80% (blue) for 1 h at 37°C. **(B)** ζ-potential distribution N(ζ) of gold NPs before (black) and after exposure to 80% human plasma (red) for 1 h at 37°C. The change in size or surface charge of NPs is due to the PC formation. Data acquired with Malvern Zetasizer Nano-ZS.

An example of the characterization of NPs with DLS before and after corona formation is represented in Figure 3A.

#### 4.4.2 Zeta potential

The surface charge of NP-conjugates, expressed as zeta (ζ) potential, is commonly measured by laser Doppler anemometer (LDA), which measures the electrophoretic mobility of charged NP-conjugates suspended in a solution toward the electrodes through an electrophoresis experiment. (Pons et al., 2006; Cho et al., 2013).The resulting ζ-potential value reflects the colloidal stability status of NP-conjugates. Particles with zeta values ≥ (+/−) 30 mV are considered highly stable but particles with zeta values ≤ (+/−) 30 have the potential to form agglomeration. (Sapsford et al., 2011; Cho et al., 2013). Many factors can affect the measurement of ζ-potential, including pH, ions concentration in the solution, and temperature. (Brant et al., 2005; Xu et al., 2018). For example, when the suspension medium is alkaline, the negatively charged NPs acquire a more negative value and *vice versa*. (Xu et al., 2018). Therefore, all information on the NP suspension medium must be precisely described in the SOP of the ζ-potential. (Brian and Kirby, 2004; Kirby and Hasselbrink, 2004). A detailed protocol on how to prepare NP samples and set up SOP for ζ-potential measurement is available at the website of the NCI-NCL (Cancer, 2024) and at the website of the EUNCL (Caputo, 2016). To achieve precise and high-quality measurements of NP-HC complexes, it is imperative to adhere to the following recommendations:

1. **Sample preparation:** Ensure complete dispersion of NPs in the solution to avoid aggregation, which can affect zeta potential measurements. Use appropriate dispersants compatible with the NP-HC complexes (e.g., NaCl) to maintain stability and ensure conductivity. The sample must be freshly prepared before the measurement. While alternative buffers like PBS are permissible for use, it is essential to note that their utilization may pose a risk of electrode damage.
2. **Measurement protocol:** Follow standardized protocols for zeta potential measurements (provided by the manufacturer), including appropriate sample dilution to ensure the measurement falls within the instrument’s dynamic range. Perform multiple measurements on each sample to account for variability and ensure reliability.

An example of characterizing the change in the surface charge of NPs after corona formation is represented in Figure 3B.

#### 4.4.3 Nanoparticle Tracking Analysis

Nanoparticle Tracking Analysis (NTA) is a complementary technique to examine the dynamic behavior of NPs suspended in liquid environments. This method employs complex imaging technology to capture and analyze the trajectories of individual particles in real time. Instead of using light scattering as DLS, it uses a CCD camera and augmentation to account for the Brownian motion of NPs. Using video microscopy and specialised tracking software, the recorded mean squared displacement of the particle over time correlates to the diffusion coefficient, which is in turn inversely proportional to the particle size. (Gallego-Urrea et al., 2011). This technique is often used for the characterization of organic NPs such as extracellular vesicles (EVs), and can be used for other types of NPs in different environments. (Vestad et al., 2017; Soliman et al., 2024). In fact, the adsorption of proteins onto the surface of NPs and the formation of the PC on its surface increases their tracked size. This increase is measured due to a decrease in the diffusion speed of the particles, observable through PTA. (Di Silvio et al., 2015). A detailed protocol on how to use PTA was published by the European Nanomedicine Characterisation Laboratory (EUNCL). (Maguire, 2018). To achieve precise and high-quality measurements of NP-HC complexes, it is imperative to adhere to the following recommendations:

5. **Sample preparation:** Maintain a low NP concentration to avoid overlapping trajectories and ensure that individual particles can be tracked accurately. Proper isolation helps in analyzing the true behavior of each NP. Keep environmental conditions like temperature and vibrations strictly controlled, as they could affect the calculation of the particle size. Consistent temperature prevents changes in fluid viscosity affecting mobility, and a vibration-free setup reduces noise, enhancing the clarity of recorded movements
6. **Measurement conditions:** Adjust the focus, lighting, and frame rate to maximize contrast and capture better the movements of NPs. This is important for accounting the changes produced by the HC on the NP size. Utilize advanced tracking software and validate results through repeated trials to ensure accuracy and repeatability in the data analysis.

**FIGURE 4:**
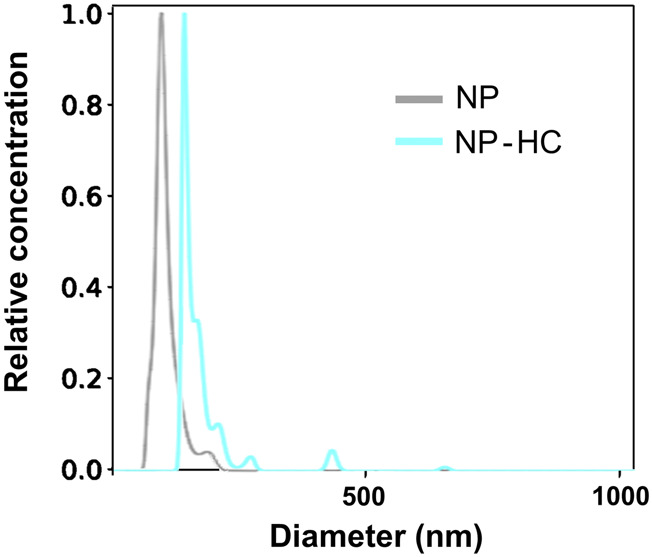
Characterization of the hydrodynamic size of silica NPs by NTA before (gray) and after exposure to 80% human plasma (blue) for 1 h at 37°C. The observed change in size is due to the PC formation. Data was acquired with Malvern Nanosight NS300.

An example of the characterization of NPs with PTA before and after corona formation is represented in Figure 4.

#### 4.4.4 Differential Centrifugal sedimentation (DCS)

**FIGURE 5:**
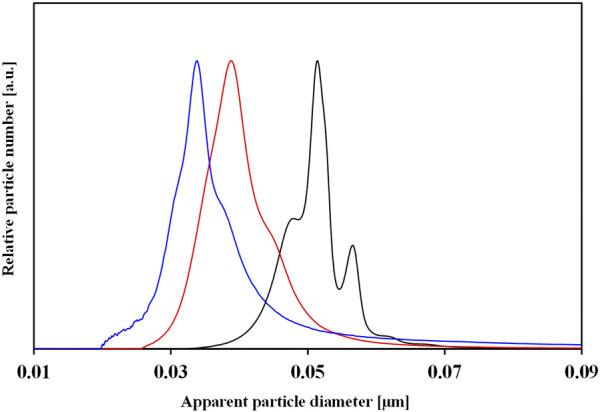
DCS normalized number size distributions of pristine gold NPs (black color) and HC-NP complexes isolated by centrifugation upon incubation with 10% (red color) and 80% (blue color) of human plasma. The change in particle size of NPs is due to the difference in PC composition.

DCS is a benchtop technique commonly used to measure the particle size distribution based on the sedimentation time through a gradient exposed to a centrifugal force. Typically the gradient is made of sucrose, but other gradients such as Optiprep® can be used. The time of sedimentation depends on the size and density of the particles. Thus, a small difference (as little as 2%) in the overall particle density due to the particle conjugation or PC formation can affect their sedimentation time (Murdock et al., 2008). DCS measures the changes in the sedimentation time and then correlates it to the particle size (usually, reported as apparent particle diameter) using Stoke’s law. (Takahashi et al., 2008). DCS can analyze a wide range of particle sizes, ranging from 5 nm to 75 μm (Murdock et al., 2008) with a high-resolution separation and detection of a small percentage of particle populations within a polydisperse colloidal sample, which cannot be detected by other techniques. On the other hand, the measurement method can take longer time than other techniques, especially for very small NPs. An example of the change in the particle size because of the PC formation is represented in Figure 5, where the size of pristine NPs (no corona) shifted due to the change in the overall density. The instrument is accurate in obtaining the absolute size of particles with a known density only. However, a core-shell model allows measuring the thickness of the coating and the density of the material (Perez-Potti et al., 2021). A detailed protocol on how to set up an SOP and perform a measurement is provided below.

1. Determine the best aqueous sucrose gradient that suits particle material density. For particles with a density <1.3 g/cm^3^, prepare a gradient concentration of 2% and 8% (w/w). For particles with density ≥1.3 g/cm^3^, prepare a gradient concentration of 8% and 24% (w/w).
2. Set up SOP in DCS software by updating the existing or creating a new one according to the properties of particles under investigation (material density, size) and standard calibration NPs (material density, size, index). Set up a broad range of size measurements to detect any large change in particles size such as aggregation. Save your SOP and exit.
3. Set up the centrifugal speed of the disk manually according to the particle size and density. A higher disk centrifugal speed should be used for NPs with a lower core material density. For example, centrifugal speeds of 14,000, 18,000, and 22,000 rpm are recommended for sizes of around 100 nm of gold, silica, and polystyrene NPs, respectively.
4. Inject the first portion of sucrose gradient into the disk and press ‘start’ to launch the disk rotation. Monitor on the temperature and speed through the screen located on the DCS case. Ensure that the temperature is less than 30°C and the speed on the screen matches the one you set manually.
5. Once you are sure that the DCS is performing well and the disk spinning is at full velocity, start injecting the step gradient following the manufacturer’s procedure. The gradient is built from nine sucrose solutions that gradually change the sucrose concentrations reversely.
6. Finish the gradient with the addition of 0.5 mL n-dodecane to minimize the evaporation of water and extend the lifetime of the gradient.
7. Allow the gradient to set and equilibrate for 30 min before use.
8. The gradient is considered stable if the results of running at least two test samples are identical. If not, the test should be repeated again after an additional 15 min or a new gradient should be prepared. To change the gradient, the instrument must be stopped, emptied, and a new gradient formed. Note that the sucrose gradient slowly degrades due to molecular diffusion. Consequently, make sure to change the sucrose gradient after 20 injections of given NPs, after 6 h of measurement, or when the gradient is no longer steep enough to maintain stable sedimentation.
9. Prepare 100 μL of given NPs or isolated NP-HC complexes preferably in water at a concentration of 0.05–1 mg/mL. The right particle concentration is determined based on the NPs’ refractive index and light adsorption. For example, particle concentration of 0.05–0.1 mg/mL is identical in the case of gold and polystyrene NPs because of their high light absorption, but more particle concentration (up to 1 mg/mL) must be used in the case of other types of NPs (e.g., silicon dioxide NPs) that have lower absorption of light.
10. Run the sample following the DCS software instructions. First, inject 100 μL of the calibration standard NPs and once the data of the standard NPs are collected, inject 100 μL of pre-prepared sample.
11. To ensure reproducibility, three measurements of given NPs should be recorded, and the absolute size value is represented as an average of three measurements with standard deviation.

#### 4.4.5 Transmission Electron Microscope (TEM)

**FIGURE 6:**
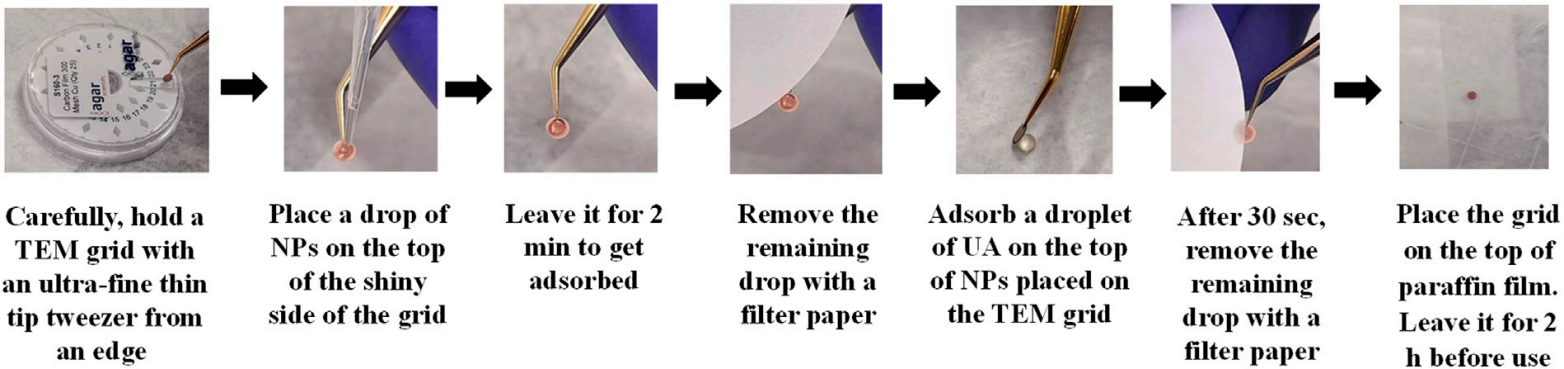
Illustration of the negative staining steps of NPs after corona formation.

TEM is a frequently used technique to characterize the size and shape of the NPs along with the shell size of their conjugates. (De Carlo and Harris, 2011; Kokkinopoulou et al., 2017; Xu et al., 2018). The measurement relies on the different contrast produced by the particles placed on the top of a TEM grid with a wave of accelerated electrons to form a highly magnified final image. Imaging of the conjugates attached to the particle core can be facilitated by staining the particles with a contrast agent such as uranyl acetate. Although the drying step of the particles sample on a TEM grid is typically required for TEM analysis, researchers found that it could alter the size of the conjugate layer attached to the particle core (Douglas et al., 2009; Steinmetz et al., 2009; Steinmetz et al., 2010; Thobhani et al., 2010) or induce particle aggregation. Therefore, it is recommended the sample of NPs-bioconjugates be super clean and sufficiently diluted before TEM measurement. A detailed protocol on how to prepare a TEM samples is available at the website of the Nanotechnology Characterization Laboratory (NCL) - National Cancer Institute, (Cancer, 2024), and an example is shown in Figure 6.

##### 4.4.5.1 Protocol for visualizing and determining the thickness of PC shell (negative staining technique)

**FIGURE 7:**
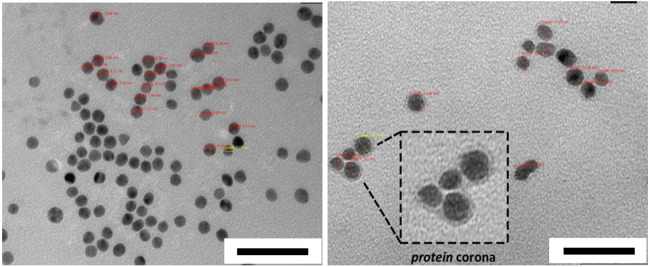
Negative staining TEM images of AuNPs before (left) and after HC formation obtained from human serum (right). Scale bars correspond to 50 nm. The figures are adapted and modified with permission from Chantada-Vázquez, et al., under Creative Commons Attribution 4.0 International License (CC BY 4.0).

1. Dilute the NP-HC complexes to a total concentration of 1–10 μg/mL in water.
2. Place 4 μL of 2% uranyl acetate on the top of a small piece of paraffin film placed inside a fume hood.
3. Hold a carbon-coated TEM grid with an ultra-fine thin tip tweezer.
4. Place a drop of 4 μL NP-HC complexes on the top of the shiny carbon side of the TEM grid and then leave it for 2 min to get adsorbed. After that, remove the remaining drop with a filter/blotting paper.
5. Put the tweezer holding the TEM grid close to the uranyl acetate droplet to get adsorbed on the top of the particles and then leave it for 30 s. After that, remove the remaining drop with filter paper.
6. Immobilize the TEM grid on the top of a stretched paraffin film inside a plastic dish and then leave it for 2 h to dry. Ensure that the carbon side where you placed the particles is upwards.
7. Place the grid in TEM and carry out the standard operating procedure analysis according to the manufacturing instructions.
8. Collect TEM images at different magnifications. Ensure that the number of particles are enough for qualitative analysis. An example of negative staining TEM image of PC shell is shown in Figure 7.
9. Analyze the acquired images using ImageJ. The thickness of the organic shell around the NPs (including the PC layer) can be determined as the total radius of the NP minus the inorganic core radius. By knowing the thickness of pre-existing organic shell (before corona formation) of the pristine NPs, we can determine the thickness of the PC layer. (Xu et al., 2018).

#### 4.4.6 Agarose gel electrophoresis

**FIGURE 8:**
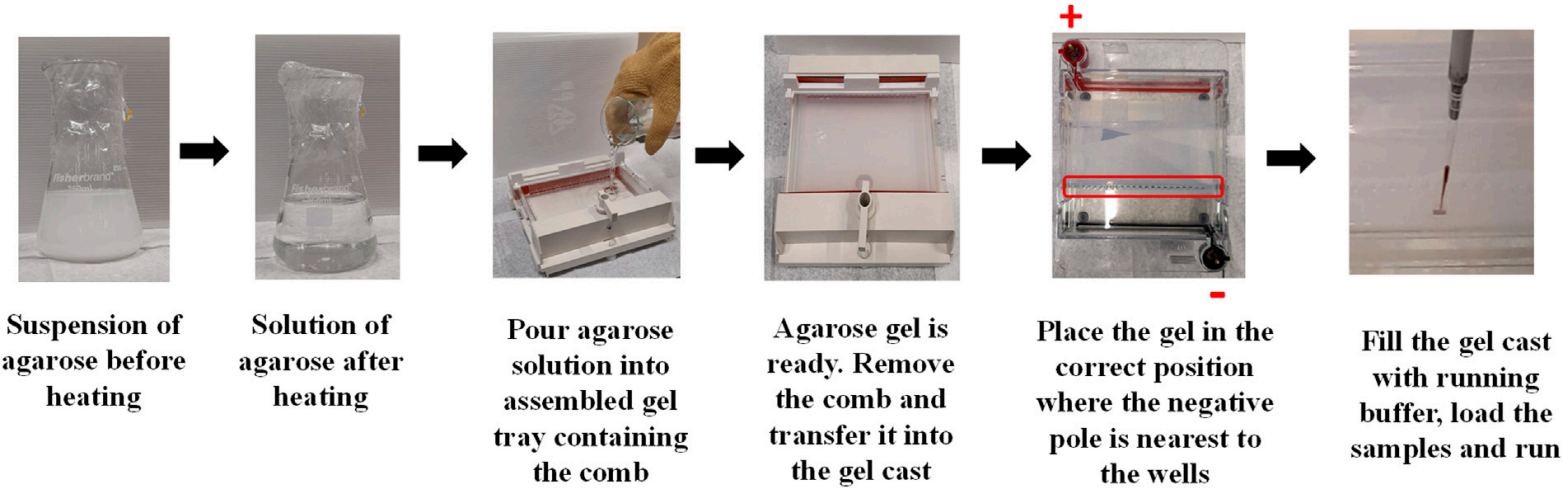
Illustration of the steps involved in the preparation of agarose gel and loading the particle sample.

Agarose Gel electrophoresis (AGE) is one of the most common and cost effective separation techniques used for the characterization of NP-conjugates that can provide information on change of size or surface charge. AGE involves applying an electric current across a gel containing the NPs of interest. The gel is usually described in term percentage and commonly prepared in a concentration between 0.5%–3% (wt/wt) of Agarose powder. It consists of a network of polymer bundles with a pore size of 100–300 nm that allows the NPs to travel through the gel in a different direction based on their size, shape, and charge under the applied voltage. In this way, researchers use it to purify different types of NPs after conjugation reactions/exposure to proteins (e.g., PC) (Pellegrino et al., 2008; Mastroianni et al., 2009; Bücking et al., 2010; Welsher et al., 2015) or to distinguish and separate different shapes of NPs (e.g., nanords from spherical NPs). (Huo, 2011). Here, the gel band containing the NP-conjugates of interest is cut from the agarose gel, transferred into a dialysis membrane with a proper molecular cutoff, and the gel runs for an additional 10 min to extract the particles from the gel band. AGE is also used to confirm the bioconjugation of NPs with different biomolecules, including proteins, as the linked biomolecules to the surface of NPs can affect their hydrodynamic size and surface charge, resulting in delay or increase in the migration speed of the NP-conjugates through the gel. (Bartczak and Kanaras, 2011; Colombo et al., 2016; Xu et al., 2018). Moreover, it can be used to check the stability of NPs as those with low stability can aggregate under the applied electric field. Furthermore, electrophoretic mobility of NPs can be quantitatively explained by a model based on the Henry formula, as reported by Hanauer et al. (Hanauer et al., 2007) For preparation and use of the agarose electrophoresis, a comprehensive protocol is described in Figure 8 and provided below.

##### 4.4.6.1 Agarose gel preparation

1. Prepare Tris-Acetate-EDTA (TAE) or Tris-Borate-EDTA (TBE) buffer to produce and run the gel. Buffers can be first prepared in concentrated solution (10x) and then diluted (1x or 0.5x) when ready to add agarose and cast the gel. For example, a 10X buffer can be prepared by dissolving 108 g of Tris-Acetate (or 55 g of Tris-Borate) in 750 mL distilled water placed in a 1 L glass bottle. To the same bottle, add 7.5 g of EDTA and dissolve it completely. Then, adjust the total volume to 1 L using distilled water. Note the buffer should have a final pH of ∼8.3 and thus, no pH adjustment is required.
2. Determine the best percentage of the agarose gel that suits the core size of given particles. It is usually recommended to use a percentage of 2%, 1% and 0.5% for particles with core size <20 nm, >20 nm, and >60 nm, respectively. The percentage measurement is a weight/volume solution. For instance, 1% gel would be 2 g of agarose in 200 mL of a buffer.
3. Weigh out the agarose powder in a conical flask; ensure that you select a flask with enough room for the bubbling solution that results when you heat it.
4. Measure out the volume of the working buffer (TAE or TBE) and add it to the conical flask containing the agarose powder. Then, cover the mouth of the flask/beaker with plastic wrap and swirl it for a few seconds.
5. Place the flask inside the microwave and heat it for 1 min. Then, take it out and swirl it to mix well.
6. Place the flask again inside the microwave and heat it for another 1 min. Then, take out and swirl it to mix well.
7. Place the flask again inside the microwave and heat it until the solution begins to boil. Keep an eye on the heating solution to avoid over-boiling.
8. Use heat-resistant gloves to take the flask out and very gently swirl it.
9. Pour the agarose solution into the gel cast tray containing the gel comb. Avoid producing bubbles, especially around the gel comb. However, if you do see bubbles, pop them using a pipette tip.
10. Cover the gel cast with tissue paper to avoid dust and leave it for some time to set. The time required for the gel to get ready depends on gel percentage and surrounding temperature. Higher agarose concentration or cooler environments will allow the gel to set faster than lower agarose concentration or warmer environments. It usually takes at least 30 min to set.
11. Carefully, remove the combs without splitting the wells.
12. Transfer the gel into the electrophoresis tank and fill it with 1x or 0.5x TEA to the maximum limit indicated on the side of the tank. Ensure that the gel is placed in the correct position; the negative pole (black in colour) is located at the bottom where the wells are nearest. Then, the gel will run from a negative pole to positive pole (red in colour, located at top of the gel).
13. The gel is now ready for samples loading.

##### 4.4.6.2 Sample loading and gel running

**FIGURE 9:**
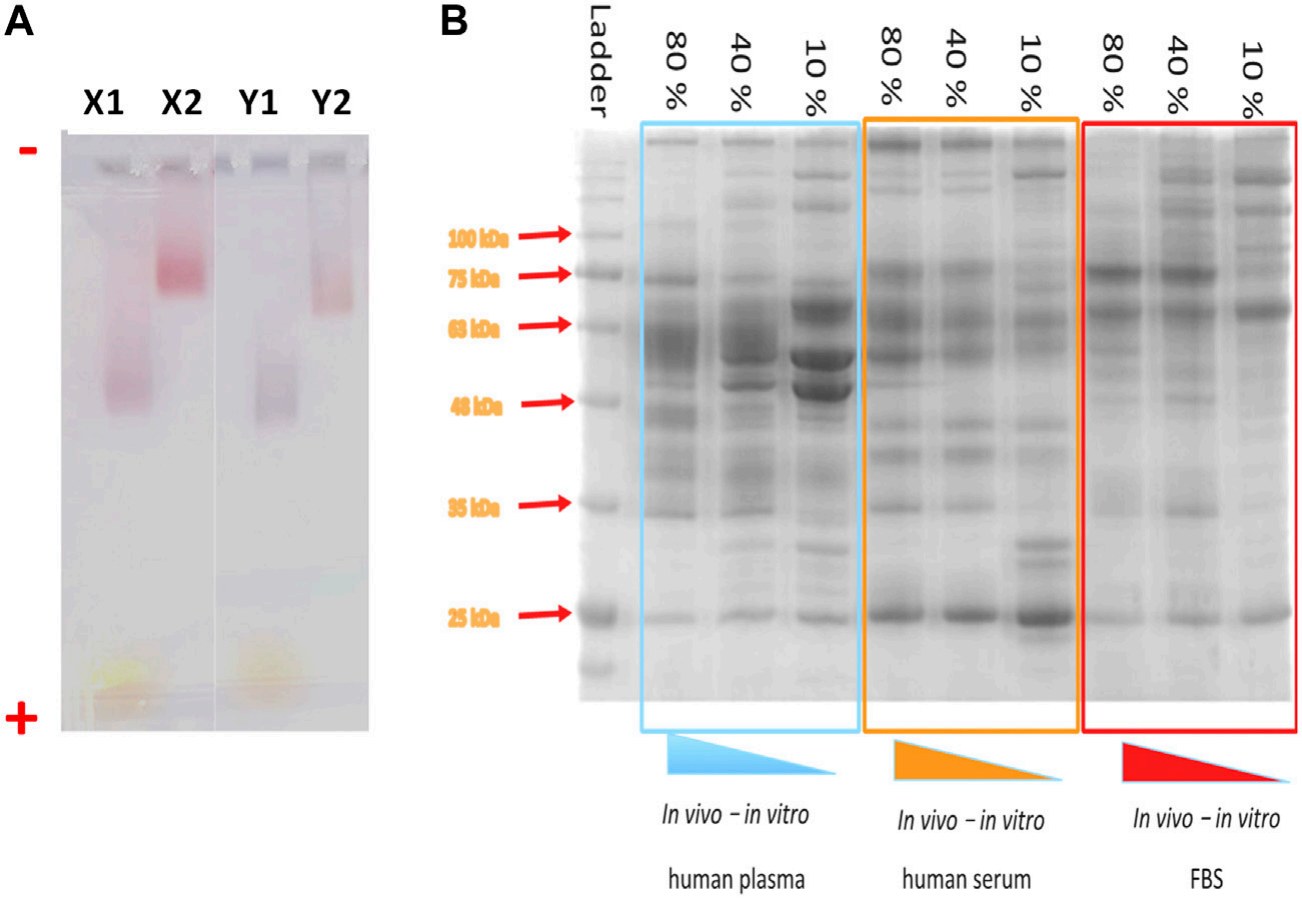
Electrophoresis gels for the characterization of NP-HC complexes. **(A)** Migration of spherical gold NPs (X) and gold nanorods (Y) through 1% agarose gel after 1 h gel electrophoresis at 10 V cm^−1^. The gel lanes show the migration of negatively charged NPs (no corona–X1 and Y1) and after corona formation (X2 and Y2). The NPs were incubated in 80% (v/v) of plasma and subjected for 3 washes cycles by centrifugation. (−) assigned to the negative electrode and (+) assigned to the positive electrode. **(B)** SDS-page of protein absorbed to IONPs’ surface after incubation with different concentrations of human plasma (left), human serum (middle) and fetal bovine serum (Right).

14. Mix your samples with a gel loading buffer (e.g., orange g or 10% v/v glycerol) to enable the samples to sit and stay in the agarose wells. For example, add 5 μL of gel loading buffer into 20 μL of your concentrated samples.
15. Load your samples slowly into the well using a pipette and avoid stabbing the gel or getting the samples back out of the wells.
16. Place the lid on the tank in the right position of the poles and then connect it up to a power supply.
17. Choose an appropriate voltage (V) and running time. It is recommended to use a voltage of 100 V for 1 h for all gels percentages (0.5%–3%). Reducing the voltage will decrease the migration speed of the particles through the gel. However, avoid using voltage >100 V as this can heat the working buffer and melt the gel slightly.
18. Finally, image your gel using a camera. An example of the characterization of NPs before and after corona formation is represented in Figure 9A.
19. Electrophoretic mobilities are quantitatively explained by a model based on the Henry formula.

### 4.5 PC visualization and identification

#### 4.5.1 SDS-PAGE

**FIGURE 10:**
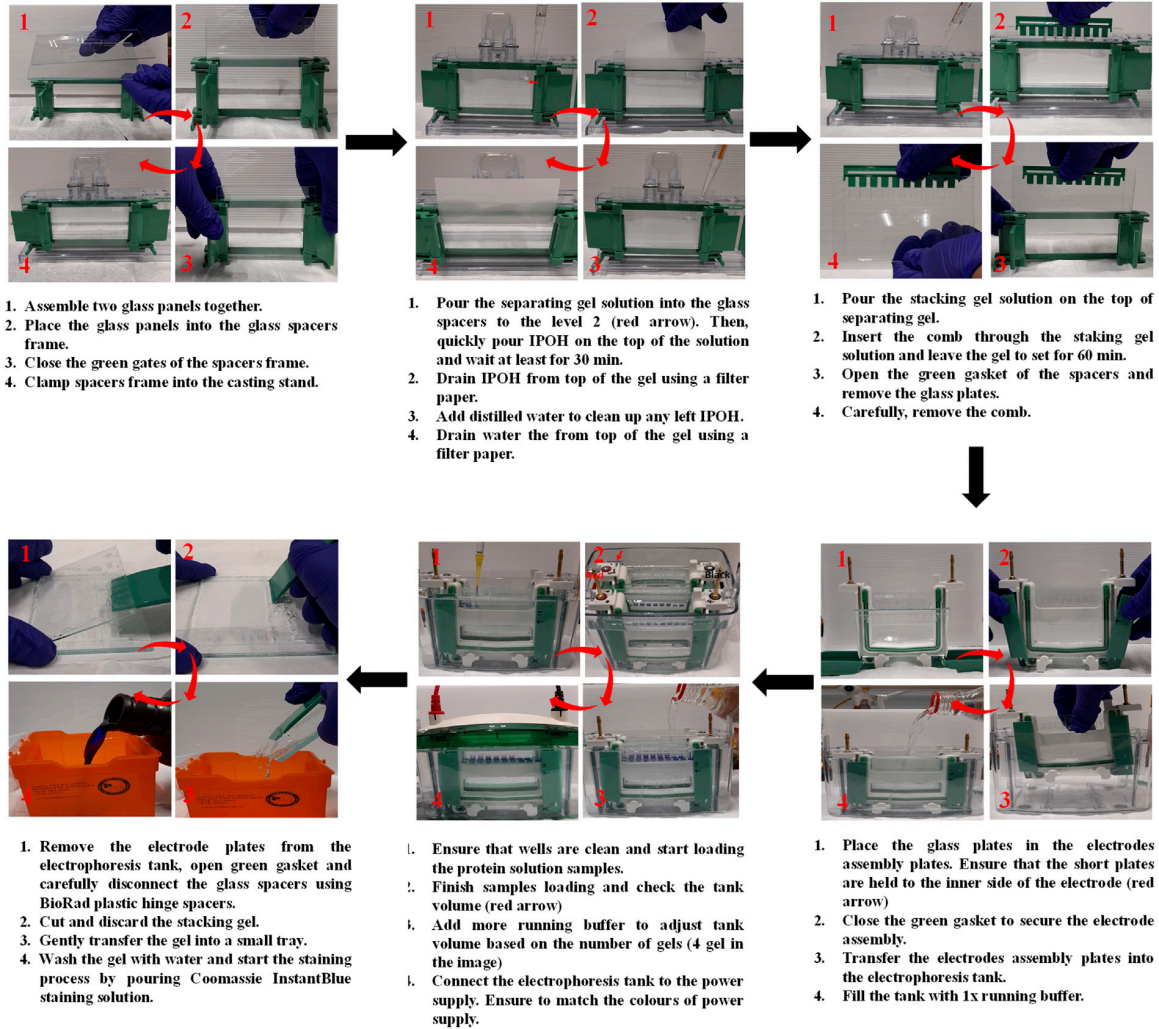
Illustration of the steps involved in the preparation of SDS-PAGE, sample loading and gel staining.

SDS-PAGE (sodium dodecyl sulfate - polyacrylamide gel electrophoresis) is a standard technique for the separation and characterization of proteins (Manabe, 2000; Winzen et al., 2015; Chen et al., 2017), sensitive and capable of detecting a low concentration of proteins (as little as ∼ 1 ng). and distinguish between proteins of a small difference (as little as a difference of 10 residues) in their peptide chains. The PAGE gels are created by the polymerization of acrylamide to produce a mesh-like matrix suitable for the separation of proteins. The mobility of the proteins through the gel matrix is proportional to their molecular weights, as small proteins migrate fast through the gel matrix while large proteins stay at the top inside the gel. Before running SDS-PAGE, proteins are regularly treated with dithiothreitol (DTT) along with sodium dodecyl sulfate (SDS) at boiling conditions. The role of DTT is to cleave the disulfide bonds between the cysteine residues of proteins and prevent the re-formation of these disulfide bonds. SDS promotes protein unfolding by binding to its backbones and generates negatively charged polypeptides, which will be separated based on chain length. This method works well for NP-corona complexes because the resulting negative charge gained by binding SDS to the backbone of protein polypeptides is usually much greater than the charge on the native protein, and thus they can be easily detached from the NP surface under the applied electrophoretic conditions. The proteins resolved in the gel can be visualized as a series of separated bands with one of the already established staining methods that give information about proteins’ types based on their molecular weights (Figure 9B). Gel can be stained with conventional staining solutions, such as blue coomassie, silver staining or fluorescent staining. Typically, the choice of the staining should be chosen based on the staining sensitivity and dynamic range of the staining. For example, blue comassie has a detection limit around 10 ng, depending on the proteins and it is usually the preferential choice due to the rapid protein band detection and high dynamic range of intensity, while silver staining offers a higher staining sensitivity (down to 1 ng) but low dynamic range. A detailed protocol on how to prepare PC samples and run SDS-PAGE is provided below and described in Figure 10.

##### 4.5.1.1 Preparation of buffer solutions

Prepare the buffers required to prepare and run PAGE gel as follows:

1. **0.4 M Tris/HCl solution, pH 6.8 (Tris/HCL 6.8)**: Dissolve 60.57 g of Tris in 800 mL of distilled water. Adjust the pH to 6.8 with 1M of HCl, and then adjust the volume to 1 L with distilled water. The solution can be stored at RT and used within 1 year.
2. **1 M Tris/HCl solution, pH 8.8 (Tris/HCL 8.8)**: Dissolve 121.14 g of Tris in 800 mL of distilled water. Adjust the pH to 8.8 with 1 M HCl and then adjust the volume to 1 L with distilled water. The solution can be stored at RT and used within 1 year.
3. **Running buffer (10x)**: In 1 L glass bottle, dissolve 30.3 g of Tris/base in 800 mL of distilled water. Then, add 144.4 g of glycine and 10 g of SDS to the same bottle. After complete dissolution of all chemicals, adjust the volume to 1 L with distilled water. The solution can be stored at RT and used within 1 year. To run SDS-PAGE, dilute the 10x of running buffer to 1x.
4. **10% (w/v) SDS**: Dissolve 5 g of SDS in 30 mL distilled water with gentle stirring to avoid foams. Then, adjust the volume to 50 mL with distilled water. The solution can be stored at RT and used within 1 year.
5. **10% (w/v) APS**: Dissolve 1 g of APS in total volume of 10 mL using distilled water. Aliquot the stock and store it −20°C. Defrost an aliquot before use and store at 4°C for no longer than 1 week.
6. **Sample loading buffer (3x)**: The buffer contains 187.5 mM Tris-HCl, pH 6.8, 6% (w/v) SDS, 30% (v/v) glycerol, 0.15 M DTT, 2% β-mercaptoethanol, and 0.03% (w/v) bromophenol blue. The loading buffer can be stored at RT and used within 1 year.

##### 4.5.1.2 Gel preparation or casting the gel

7. Assemble the glass plates into the glass spacers and then place them in the gel casting frame.
8. Determine the percentage of gel you need to separate your proteins based on their molecular weight. For example, use 5%–8%, 5%–10%, or 5%–12% gels to separate proteins of molecular weights of 120–250, 40–120, or 15–40 kDa, respectively.
9. Prepare ‘separating gel’ with a suitable percentage (step 7) using Table 1. As an example, in the following section we introduce the steps for the preparation of 5%–10% gels.
10. **10% Separating gel solution** (**preparation of 2 gels**): Freshly prepare a solution containing 3.6 mL of distilled water, 3.7 mL of M Tris-HCl (pH 8.8), 2.5 mL of acrylamide, 0.1 mL of 10% SDS, 50 μL of 10% APS.
  - Mix the components and then add 10 μL of TEMED.
  - Finish the solution preparation by mixing the whole solution for the resolving gel.
  - Quickly pour the solution into the glass plates to level 2 (third-fourths of the total volume) before the top of plates.
  - Place a layer of isopropyl alcohol (IPOH) over the top of the gel to prevent meniscus formation in the gel.
  - Leave it to set for at least 30 min at RT.
  - Drain the isopropyl alcohol from top of the gel.
  - Add distilled water and drain it to remove any remaining alcohol using a piece of filter paper. Repeat this step twice.
11. **5% stacking gel solution** (**preparation of 2 gels**): Freshly prepare a solution containing 2.8 mL of distilled water, 1.6 mL of M Tris-HCl (pH 6.8), 0.5 mL of acrylamide, 50 μL of 10% SDS and 50 μL of 10% APS.
  - Mix the components and then add 10 μL of TEMED.
  - Finish the solution preparation by mixing the whole component for the stacking gel.
  - Quickly pour the solution into the glass plates on the top of separation gel and then insert a comb through the stacking gel solution. Choose the comb that can hold enough volume of your sample. Combs of 10 and 15 wells can hold up to 30 μL and 20 µL of a prepared sample, respectively.
  - Leave it to set for at least 60 min at RT.
12. Remove the comb and place the glass plates holding the gels in the electrodes assembly plates, where each gel is positioned on each side of the electrode plate.
13. Close the green gasket of the electrode plates and then transfer them into the electrophoresis tank.
14. Fill the tank with 1x running buffer, filling first the chambers between the electrodes plates to check for any leaking. Adjust the amount of buffer based on the number of gels you will be running, 2 or 4 gels mark indicated on the tank. If you are running just one gel, place a buffer dam on the other place of the electrode plate. Ensure that the electrode plates are placed in the correct position by matching the electrodes colours of the electrode plates and electrophoresis tank.

**TABLE 1:** Quantities for each of the chemicals needed to cast two PAGE gels.

Solution components	Mw of proteins (kDa)				
	120–250	40–120	15–40	<15	
	Separating gel				Stacking gel
	8%	10%	12%	15%	5%
Deionized water (mL)	4.1	3.6	3.1	2.2	2.8
1 M Tris/HCL 8.8 (mL)	3.7	3.7	3.7	3.7	-
0.4 M Tris/HCL 6.8 (mL)	-	-	-	-	1.6
Acrylamide	2	2.5	3	3.7	0.5
10% SDS	0.1	0.1	0.1	0.1	0.05
10% APS	0.05	0.05	0.05	0.05	0.05
TEMED	0.01	0.01	0.01	0.01	0.005

##### 4.5.1.3 Sample preparation, loading and running

15. Dilute the NP-HC complexes in 3x of sample loading buffer with a ratio of 2:1, respectively.
16. Place the samples in a thermoshaker and heat the mixture at 95°C for 5–10 min.
17. Load the solution of NP-HC complexes into the gel wells. Ensure that each sample is loaded separately from the other to avoid contamination.
18. Load 1–3 μL of a ladder protein standard of known molecular weights into one well.
19. Run the gel at 120 V for 1.5–2 h or until blue front dye reaches the bottom of the gel.

##### 4.5.1.4 Staining and destaining the Gel

20. Remove the electrode plates from the electrophoresis tank.
21. Open green gaskets and remove the glass plates from the electrode plates.
22. Carefully disconnect the glass spacers using BioRad plastic hinge spacers and gently transfer the gel into a small tray.

**a) Coomassie blue staining**

23. Wash the gels with 20 mL of distilled water for 5 min while shaking gently. Pour off the water, and wash it again two more times.
24. Pour off the water, add 20 mL of Coomassie InstantBlue staining, and stain the gels for 1–2 h with gentle shaking.
25. Pour off the staining solution, and add 20 mL of distilled water with gentle shaking for 30 min to destain the gels.
26. Pour off the water, and add 20 mL of distilled water with gentle shaking for 30 min.
27. Repeat the previous step with gentle shaking until the gel is visibly destained.
28. Pour off the water. Dry the gel and place it on a UV glass film for imaging.

**b) Silver staining**

General comments:

- The volumes and times for this protocol have been adapted following the manufacturer’s instructions (Cosmo Bio). We recommend to optimise the conditions according to the sample.
- The protocol is for 1 PAGE gel with thickness 1 mm. For thicker gels, the protocol needs to be optimized with increased incubation times.
- A glass container should be used for the staining. If not, it is recommended to make the staining in a yellow or transparent plastic box, as the blue plastic boxes give an increased background.
- Cover the boxes with plastic foil to avoid evaporation.
- Mix all solutions in separate glass containers.
- When used, pour all solutions except the staining solution in the decant container.
- Pour the staining solution into a glass container containing 4 mL HCl to precipitate the silver. When silver is precipitated, remove the solution and place the silver precipitate in the waste bin with help of a paper tissue.
- The gel can be left in Fix I overnight if preferred.

Procedure:

23. 15 min Fix I = Destain solution
     o 25 mL MilliQ water
     o 20 mL Methanol
     o 10 mL acetic acid
24. 15 min Fix II
     o 27.5 mL MilliQ water
     o 15 mL Methanol
     o 5 mL acetic acid
     o 2.5 mL Reagent 1
25. 10 min Pre-treatment
     o 22.5 mL MilliQ water
     o 25 mL Methanol
     o 2.5 mL Regent 2
26. 5 min Wash
     o MilliQ water
27. 15 min Staining
     o 45 mL MilliQ water
     o 2.5 mL Reagent 3
     o 2.5 mL Reagent 4
     o Place staining solution in 4 mL HCl after procedure
28. 2 min Wash
     o MilliQ water
29. <5 min Develop
     o 47.5 mL MilliQ water
     o 2.5 mL Reagent 5
     o Look at the gel at all time and stop when the signal is good and background not too high
30. Stop
     o Add 2.5 mL Reagent 6 to beaker
31. 2 × 5 min Wash
     o MilliQ water

##### 4.5.1.5 Densitometry analysis on SDS-PAGE Gel

1. Download and open a software for gel picture analysis, such as ImageJ, which is available for free download at www.imagej.net
2. Open the saved image and draw a box around the protein band of interest using the rectangle selection tool, making sure it spans the whole lane’s width.
3. Go to “Analyze” in the ImageJ menu, choose “Gels,” and then press “Select First Lane (Ctrl+1)." This will create a first rectangle with the number one.
4. Click on the rectangle with number one. This will create a new rectangle. Drag this rectangle at the next lane of interest. When unclick, the rectangle will stay at the selected position. By clicking Ctrl+2, a number two will appear and the height will align automatically with the previous rectangle.
5. Repeat the previous step until you reach the last lane to analyse.
6. When you reach the last lane, repeat the same procedure but after dragging the rectangle, you should then click Ctrl+3.
7. A window with the band intensities will appear. You can select any band of interest and its corresponding area using the straight line to determine some relative protein band intensity.
8. By selecting the wand tracing tool, you will be able to click on the areas and quantify them. The “Results” window will appear, with the selected area values. You can also save the coordinates of the profile by clicking File > Save as > XY Coordinates.
9. Once performed to all the areas of interest, you can copy the values to any file.
10. To account for changes across gels and samples, normalize the band intensities to an appropriate reference (such as a loading control or a well-known standard), or compare the relative band intensity by normalizing the desired band intensity to the overall lane intensity.
11. To have consistent data on the protein band intensities under the same conditions, it is recommended to do statistical analysis over independent replicates.

##### 4.5.1.6 Determination of affinity constants between proteins and NPs

**FIGURE 11:**
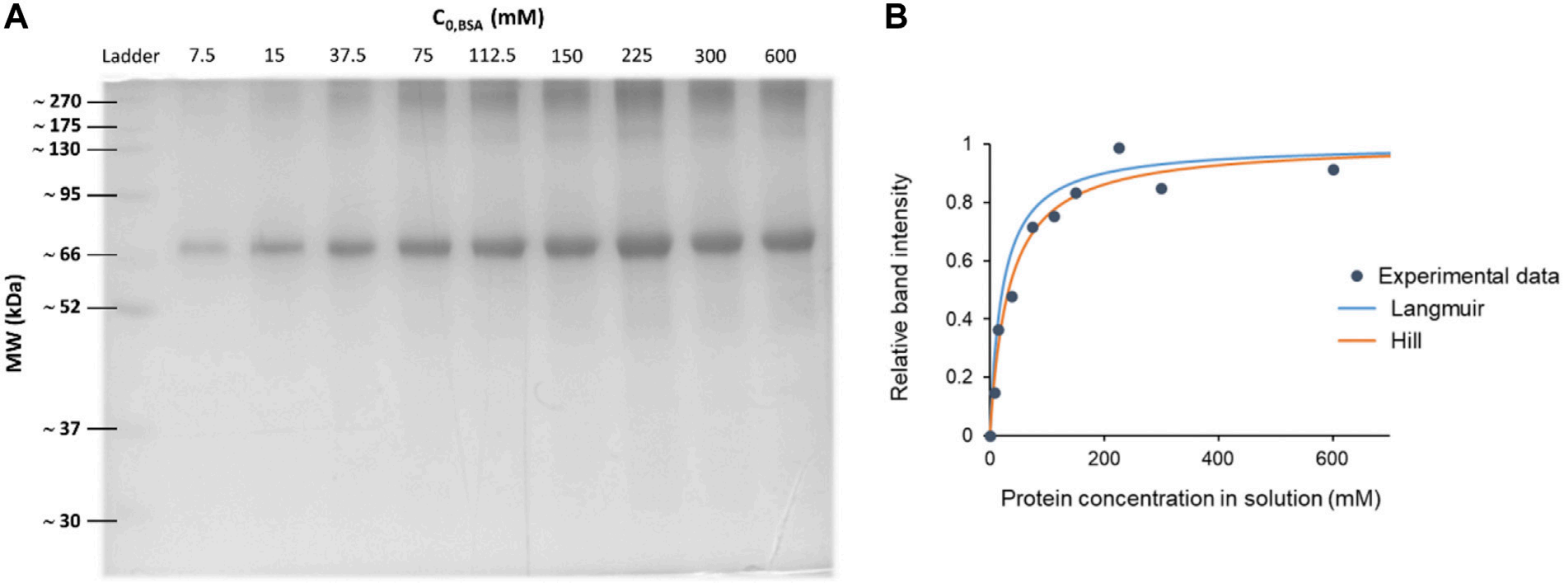
Blue coomassie stained SDS-PAGE gel and the corresponding densitometry analysis to determine affinity constants. **(A)** SDS-PAGE of Bovine Serum Albumin (BSA) coronas on 100 nm polystyrene carboxylated NPs (PSCOOH). Each lane represents a different protein concentration condition, while keeping the NP concentration constant. **(B)** Non-linear regression curves over the experimental data points, obtaining a K_D_ ≈ 0.3 µM. Note how depending on the chosen model the regression differs and fits differently the data points. A good choice of the model is crucial.

Affinity constants are key values for comprehending how proteins interact with NPs. The connection between the equilibrium concentration of protein in solution and the quantity adsorbed onto the NP surface at a specific temperature is described by adsorption isotherms. These can exhibit different types and nature, reflecting the diverse interactions between adsorbents and adsorbates. Some common models include Langmuir, Hill or Freundlich isotherms. (Syafiuddin et al., 2018). To obtain the affinity constant one can follow the steps below, and a typical example of the results is shown in Figure 11.

1. Prepare a series of samples of proteins with increasing concentrations. Starting with a low protein concentration, the protein concentration is progressively raised for subsequent samples.
2. Hard Corona and SDS-PAGE protocols are performed.
3. In order to prevent saturation effects and guarantee correct quantification, it is essential to correctly analyze the SDS-PAGE. There should not be any very light or overloaded bands. If so, one needs to repeat the procedure increasing or decreasing the NP/protein ratio, making always sure that the NP-HC complexes are colloidally stable.
4. Perform gel densitometry as described in the protocol.
5. Plot the relative protein band intensities on the *y*-axis against the initial protein in solution concentrations on the *x*-axis.
6. Non-linear regression analysis is used to fit the experimental data points to the isotherm equation. Curve fitting tools are available in a number of software packages, including GraphPad Prism (R), or one can fit the data points by introducing the equation in other software. The program will then estimate the best fit values for the dissociation constant K_D_, which is representative of the strength of the protein-NP affinity.
7. This constant is usually represented in µM–make sure units are consistent. The lower the K_D_ value, the higher the affinity between the proteins and the NPs.
8. Evaluate how well the isotherm model fits your experimental results. Consider the biological significance and context of the chosen model as well as the calculated value of the affinity constant.
9. Try to validate the results using different experimental techniques, such as DCS, fluorescence correlation spectroscopy (FCS) or bicinchoninic acid assay (BCA) (Vilanova et al., 2016). In this last technique, some NPs (like graphene oxide) can react with BCA and interfere the protein quantification. It is advisable to perform a control test with the NPs and the assay reagent but without any proteins to assess any interference.

### 4.6 PC corona quantification

#### 4.6.1 Mass Spectrometry

Mass spectrometry (MS) has become the first choice in proteomics research because of its ability to provide qualitative and quantitative analysis of complex protein samples from their peptide fragments. The basic principle of MS is to generate and separate gas phase ions of given peptides/proteins according to their mass-charge ratio (m/z) and thus display the result in the form of mass spectra representing their ion abundance *versus* m/z. There are four different types of mass analysers commonly used in proteomics analysis: i) ion trap, ii) quadrupole, iii) time-of-flight, and iv) Fourier transform ion cyclotron analyzers. They vary in design and analytical performance, and each has its own strength and weakness. Therefore, these analyzers have been combined in a hybrid system to put together their advantages in tandem mass spectrometry. An example of these hybrid instruments is liquid chromatography-tandem MS-MS (LC-MS2), which is frequently used in the identification of corona proteins. The analysis of proteome samples via MS follows either “top-down” or “bottom-up” proteomics analysis. Top-down approach is typically carried out on the whole proteins, while bottom-up approach is typically performed on enzymatically or chemically produced peptides, which can easily be ionized and fragmented in comparison to whole proteins.The latter approach is more widely used in PC analysis because the corona preparation involves multiple steps of proteins denaturation, detachment from the particle surface, digestion, and size-exclusion that end up with the production of peptides for “bottom-up” proteomics analysis. Data processing and analysis to account for the abundance of different proteins is performed using software such as MaxQuant and Perseus. (Cox and Mann, 2008; Tyanova et al., 2016). Detailed protocols on how to prepare PC samples for mass spectrometry using different approaches is provided below.

##### 4.6.1.1 In-gel Digestion protocol

In this approach, PC is isolated from the particle surface after incubation with SDS-PAGE sampling buffer containing reducing and denaturing agents under applied electrophoresis for a short period. Thereafter, the individual protein gel bands are excised from the gel and subjected to a series of chemical and enzymatic treatments to extract the peptides. The limitation of this method is the long-time tedious clean-up steps, which may lead to sample loss and affect reproducibility. (Hadjidemetriou et al., 2016).

**a) Preparation of buffer solutions**

Prepare the buffer solutions required for the sample reduction and digestion as follows:

1. Ammonium bicarbonate (NH_4_HCO_3_) buffer, pH 7.8: Prepare a stock solution of 200 mM NH_4_HCO_3_ by dissolving 15.81 g in 800 mL distilled water and then adjust the volume to 1 L. Then, dilute it 2 and 10 times to produce 100 mM and 20 mM, respectively. After that, aliquot and freeze at −20 °C. Stock solutions are stable for at least 1 month at −20 °C
2. Freshly prepare 10 mM dithiothreitol (DTT) by dissolving 1.54 mg in 1 mL of 100 mM NH_4_HCO_3_.
3. Freshly prepare 50 mM indole-3-acetic acid (IAA) by dissolving 8.75 mg in 1 mL of 100 mM NH_4_HCO_3_.
4. Mix 10 mL of acetic acid with 40 mL of methanol and 50 mL of distilled water to prepare a mixture of 10% (v/v) acetic acid, 40% (v/v) methanol and 50% (v/v) distilled water.
5. Mix 60 mL of acetonitrile (ACN) with 40 mL of 200 mM NH_4_HCO_3_ to prepare a mixture of 60% (v/v) ACN and 40% (v/v) NH_4_HCO_3_.
6. Mix 5 mL of 20 mM NH_4_HCO_3_ with 5 mL of ACN to prepare a mixture of 50% (v/v) NH_4_HCO_3_ and 50% (v/v) ACN.
7. Mix 50 mL of ACN with 5 mL of formic acid and adjust the volume to 100 mL to prepare a mixture of 50% ACN and 5% formic acid.
8. Freshly prepare a mixture of 30% (v/v) ACN and 0.2% (v/v) Trifluoroacetic acid (TFA) by mixing 3 mL of ACN with 0.2 mL of TFA and then adjust the volume to 10 mL.
9. Prepare 50% ACN by mixing 5 mL ACN with 5 mL distilled water.
10. Freshly prepare a stock solution of 1% TFA by mixing 0.1 mL of TFA with 9.9 mL of distilled water. Then, dilute it 10-folds to get 0.1% TFA.
11. Freshly prepare a mixture of 0.1% TFA and 5% CAN by mixing 1 μL TFA with 0.5 mL ACN and then adjust the volume to 10 mL.

**b) HC isolating and in-gel trypsin digestion**

12. Follow the previous protocol (2.5.1) of SDS-PAGE gel preparation and sample loading.
13. Run the gel at 120 V for 10 min at RT or until they enter into the separation gel (while the NPs will remain in the gel well or in the separation gel)
14. Wipe down enough space on a lab bench with an ethanol/water moistened lint-free cloth.
15. Transfer the glass plates to the clean bench surface and carefully disconnect the glass spacers using BioRad plastic hinge.
16. Cut around 1 cm of the bands from the gel using a clean cutter and then chop each gel slice into small pieces or roughly 2 mm. (Paulo et al., 2013).
17. Transfer the gel pieces into 1.5 mL low binding microcentrifuge tube previously treated with 0.5 mL of ACN.
18. Add 300 μL of freshly prepared mixture contains 10% (v/v) acetic acid, 40% (v/v) methanol and 50% (v/v) distilled water.
19. Using gel loading pipet tip, remove and discard the supernatant.
20. Add 70 μL of 200 mM ammonium bicarbonate to each microcentrifuge tube. Place them in a thermoshaker and heat the mixture at 37°C for 10 min
21. Spin down briefly, extract the solution and discard it (using a fresh tip for each microcentrifuge tube).
22. Add 70 μL of a mixture contains 40% (v/v) NH_4_HCO_3_ (200 mM) and 60% (v/v) ACN to each microcentrifuge tube. Place them in a thermoshaker and heat the mixture at 37°C for 10 min
23. Spin down briefly, extract the solution and discard it (using a fresh tip for each microcentrifuge tube).
24. Add 70 μL of 50 mM NH_4_HCO_3_ to each microcentrifuge tube. Place them in a thermoshaker and heat the mixture at 37°C for 10 min.
25. Spin down briefly, extract the solution and discard it (using a fresh tip for each microcentrifuge tube).
26. Dehydrate the gel pieces with 70 μL (or enough to cover) of ACN to each microcentrifuge tube. Place them in a thermoshaker and heat the mixture at 37°C for 10 min.
27. Spin down briefly, extract the solution and discard it (using a fresh tip for each microcentrifuge tube).

**c) Enzymatic digestion**

28. Add 50 μL (or enough to cover) of 10 mM DTT to each microcentrifuge tube. Place them in a thermoshaker and heat the mixture at 56°C for 60 min.
29. Spin down briefly, extract the solution and discard it (using a fresh tip for each microcentrifuge tube).
30. Alkylate free cysteine by adding 50 μL of 50 mM IAA. Allow the reaction to proceed in the dark for 30 min at RT, gently shaking.
31. Spin down briefly, extract the solution and discard it (using a fresh tip for each microcentrifuge tube).
32. Treat the gels pieces with 300 μL of 100 mM NH_4_HCO_3_. Place them in a thermoshaker and heat the mixture at 37°C for 15 min.
33. Spin down briefly, extract the solution and discard it (using a fresh tip for each microcentrifuge tube).
34. Add 300 μL of a mixture contains 50% (v/v) 20 mM NH_4_HCO_3_ and 50% ACN. Place them in a thermoshaker and heat the mixture at 37°C for 15 min.
35. Spin down briefly, extract the solution and discard it (using a fresh tip for each microcentrifuge tube).
36. Dehydrate the gel pieces with 100 μL (or enough to cover) of ACN to each microcentrifuge tube. Place them in a thermal shaker and heat the mixture at 37°C for 10 min.
37. Spin down briefly, extract the solution and discard it (using a fresh tip for each microcentrifuge tube).
38. Estimate the gel volume and add about 3x volume of freshly prepared trypsin solution.
39. Add 25–50 μL trypsin solution (1 μL stock trypsin in around 200 μL of ammonium bicarbonate 50 mM) to just barely cover the gel pieces.
40. Place the microcentrifuge tubes in a thermal shaker and heat the mixture at 37°C for at least 4 h - usually overnight.

**d) Extraction of digested Peptides**

41. Spin down briefly and transfer the digested solution (aqueous extraction) into clean 1.5 mL low binding microcentrifuge tubes labelled with the same sample name and/or number.
42. To the gel pieces, add 30 μL (or enough to cover) of freshly prepared mixture containing 50% (v/v) ACN and 5% (v/v) formic acid, vortex 1 min, sonicate 5 min, and spin down briefly. Place them in a thermoshaker and heat the mixture at 37°C for 10 min.
43. Extract the solution and add it to a clean microcentrifuge tube labelled with the same sample name.
44. Add 70–100 μL of a fresh prepared mixture contains 30% (v/v) ACN and 0.2% (v/v) TFA to each microcentrifuge tube. Place them in a thermoshaker and heat the mixture at 37°C for 10 min.
45. Repeat step 43 one more time.
46. Spin down briefly and transfer the solution (using a fresh tip for each tube) into a clean 1.5 mL low binding microcentrifuge tubes labelled with the same sample name and/or number.
47. Place the microcentrifuge tubes (open the caps) in SpeedVac. Set the temperature at 60°C, start the rotation and leave the sample to dry. This process usually takes 2–3 h.

**a) Purification and collection of the peptides for mass spectroscopy**

48. Add 100 μL of 1% TFA for each sample and sonicate for dispersion.
49. Attach a C18 tip to the end of 100 μL pipette for optimum tip-to-pipettor seal and sample aspiration.
50. Aspirate the tip in 100 μL 50% ACN in water and then discard the solvent. Repeat once.
51. Aspirate the tip in 100 μL 0.1% TFA in water and then discard the solvent. Repeat once.
52. Aspirate the tip in 100 μL of a peptides sample (step 47). To achieve higher efficiency, dispense and aspirate sample for at least 10 cycles.
53. Aspirate the tip in 100 µL of 0.1% TFA/5% ACN and discarded solvent. Repeat once.
54. Slowly aspirate the tip in 100 µL of 0.1% formic acid or 0.1% acetic acid in a 50%–95%.
55. Place the microcentrifuge tubes (open the caps) in SpeedVac. Set the temperature at 60°C, start the rotation and leave the sample to dry. This process usually takes 2–3 h.
56. Add 40 μL of 0.5% acetic acid for each sample, sonicate to disperse and check that there is sufficient protein in the sample using spectrophotometric absorbance in the aromatic region, such as A280 in Nanodrop. (Desjardins et al., 2009). Store it at −20 °C for the characterization by LC-MS2.

##### 4.6.1.2 In-Solution digestion protocol

In this approach, the corona proteins are desorbed from the particle surface using a high concentration of denaturing agents. Then, the proteins are collected by centrifugation, digested, and the peptides are extracted and processed for MS analysis. Then, the proteins are collected by centrifugation and digested with trypsin. After that, the peptides are extracted and processed for MS analysis. The critical step of this method is the desorption of corona proteins from the NP surface. Incomplete desorption can lead to misrepresentation of the PC composition, impacting the accuracy of subsequent analyses. Careful consideration of the desorbing agent and conditions ensures the reliability and validity of the obtained results by MS analysis. The reader is referred to follow a detailed protocol reported by Docter et al., 2014.

#### 4.6.2 Glyco-profiling protocol

**FIGURE 12:**
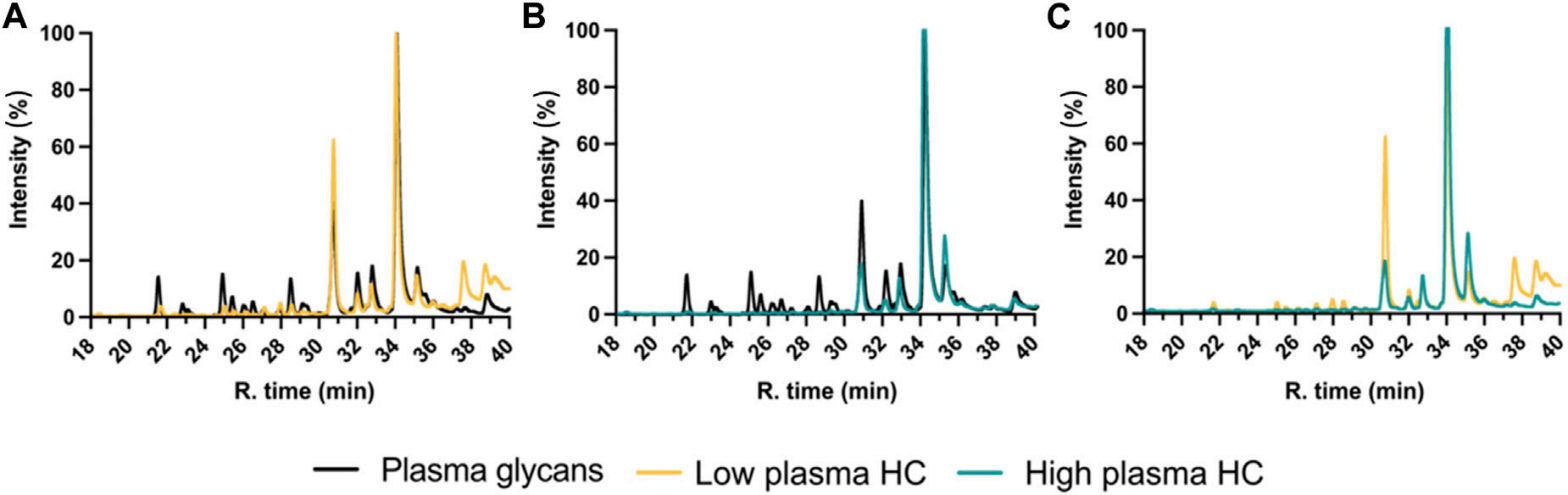
Normalized UHPLC-FLD chromatograms section ranging from minute 20 to minute 40 of procainamide labelled N-glycans. See **(A)** low and **(B)** high plasma protein hard corona on 100 nm silica NPs, compared to the glycans found in plasma, while **(C)** is a comparison between low and high plasma HC.

While most studies have focused on the protein component only, few have recently explored the role of N-glycans within the corona components. Glycosylation is a common post-translational modification for plasma proteins and plays a crucial role in a wide range of physiological processes and immune system recognition. These glycan components of the corona impact interactions between NPs and biological systems, thereby influencing critical factors such as biocompatibility, biodistribution, and cellular uptake. (Wan et al., 2015; Cai et al., 2018; Higel et al., 2019). Methods for studying N-glycans present in the corona encompass several techniques. (Wan et al., 2015; Duong et al., 2022; Clemente et al., 2022). Lectin affinity chromatography capitalizes on lectins’ specific carbohydrate-binding properties, enabling the selective isolation of N-glycans based on their affinity for immobilized lectins. (Wan et al., 2015; García et al., 2015; Clemente et al., 2022). Hydrophilic interaction chromatography (HILIC) exploits differences in N-glycans’ hydrophilicity for separation, often coupled with mass spectrometry for comprehensive glycan analysis. Glycan release and labeling involve enzymatic release of N-glycans from glycoproteins or glycolipids, followed by labelling with fluorescent or chemical tags for subsequent purification and analysis. Additionally, mass spectrometry-based glycomics offers direct analysis of N-glycans from complex biological samples, employing techniques such as matrix-assisted laser desorption/ionization (MALDI) or electrospray ionization (ESI) coupled with liquid chromatography (LC-MS). These methods facilitate the isolation and characterization of N-glycans, enabling the study of the glycan corona and its implications in biomedical research and applications. (Duong et al., 2022; Clemente et al., 2022). N-glycans are extracted from the PC using the LudgerZyme PNGaseF kit. Data analysis to quantify the abundance of different N-glycans is performed through software such as HappyTools. (Jansen et al., 2018). A protocol for glyco-profiling is provided below, and a typical example of the results is shown in Figure 12.

1. **Denaturing conditions**. NP-HC complexes are centrifuged at 18,000 RCF for 10 min to form a pellet and then resuspended in 15 μL of ultrapure water. Next, 10 μL of 10x denaturing solution containing 5% SDS and 400 mM DTT is added to each sample and mixed thoroughly. The samples are heated at 100°C for 10 min, followed by a brief vortex and centrifugation at 18,000 RCF for 10 min to eliminate any remaining NPs. To each glycoprotein-containing supernatant, 20 μL of 10x reaction buffer, 20 μL of 10% NP-40 solution, 135 μL of pure water, and 1 μL of PNGase F are added. After vertexing, the samples are incubated at 37°C overnight (14–16 h).
2. **PNGaseF treatment in native conditions**. The N-glycan release can be performed in native conditions, according to the protocol provided by Ludger LTD. After the last wash, the NPs are resuspended in 18 μL of Milli-Q water and both reaction buffer 10x and PNGase F enzyme are added. Incubation is performed for different time points at 37°C in non-shaking conditions. At the end of the incubation time, the particles are resuspended in 200 μL of PBS.
3. **Glycan clean-up**. This step is done to separate the glycans from the proteins in solution. The samples are resuspendend in 200 μL of PBS and spun down at 18,000 rcf. The pellet is discarded while the supernatant is collected and freeze dried. The dried sample is incubated with a solution 1% formic acid for 50 min at room temperature in order to hydrolyse the glycosylamine and produce the labelable reducing end. Next, the glycans from the samples are separated from the proteins by using a hydrophobic membrane-plate and a vacuum manifold. The membrane on a 96 well plate is first activated by the addition of 100 μL of methanol in the wells and by applying vacuum. The membranes are then cleaned and primed by adding 300 μL of water and applying vacuum. The collection tubes are placed under the membrane, the samples containing the glycans are added one to each well and collected by applying vacuum. The wells are washed twice with 100 μL of water and the final collected solution is freeze dried overnight.
4. **Procainamide labelling.** The samples are freeze dried ensuring that the sample dries to a small, compact mass at the very bottom of the vial. Higher temperatures than 28°C or extremes or extreme pH should be avoided as these conditions could result in acid catalysed loss of sialic acids or epimerization of the glycan reducing terminus. Once dried, 10 μL of water are added to re-dissolve glycans. The labelling is performed with the LudgerTag™ following manufacturer’s instructions. 10 μL of a mixture of acetic acid in DMSO, procainamide dye and 2-picoline borane are added to each sample. The samples are then incubated in a heating block set at 65°C and incubate for 1 h. To remove the free dye and unreacted chemicals, the samples are cleaned up using LudgerClean™ Procainamide Clean-up Plate per manufacturer’s instructions.
5. **LC analysis.** Procainamide-labelled samples and system standards are analysed using HILIC-UHPLC-FLD with an excitation wavelength of 310 nm and an emission wavelength of 370 nm. The adequate UHPLC is kept at 40°C. Solvent A typically contains a 50 mM ammonium formate buffer pH 4.4, while Solvent B is acetonitrile. To prepare the samples, 75 μL of acetonitrile is mixed with 25 μL of concentrated and labelled glycans. Then, 12.5 μL of the resulting solution is injected into the column. The samples are maintained at 4°C in the autosampler, with the oven containing the column set to 40°C. The fluorescent unit detector parameters are adjusted to excitation 310 nm and emission 370 nm. The UHPLC gradient is set according to Table 2.

**TABLE 2:** N-glycan analysis gradient. It describes a long UHPLC gradient for samples where the glycan profile is unknown.

Time (min)	% Solvent B	Flow rate (mL/min)
0	76	0.40
53.5	51	0.40
55.5	0	0.25
57.5	0	0.25
59.5	76	0.25
65.5	76	0.25
66.5	76	0.40
70.0	76	0.40

#### 4.6.3 Sialic acid quantification protocol

**FIGURE 13:**
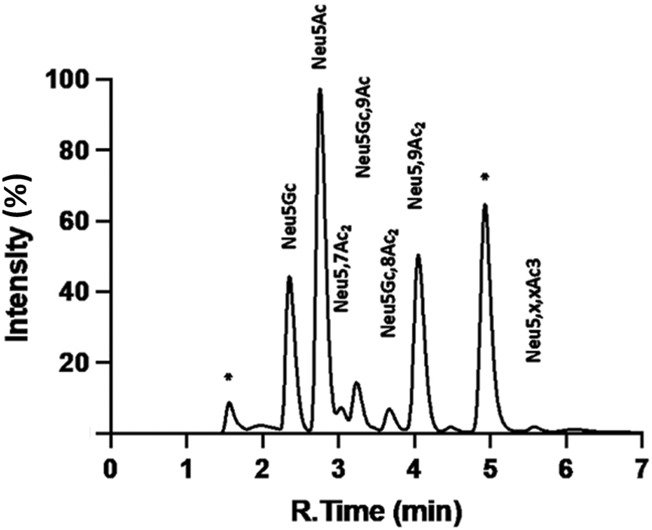
Chromatogram of DMB Labelled Sialic Acid Reference Panel run on the LudgerSep-R1 HPLC column. Peaks, from left to right, Neu5Gc; Neu5Ac; Neu5,7Ac_2_; Neu5Gc,9Ac; 5 Neu5,8Ac_2_; Neu5,9Ac_2_; Neu5,x,xAc_3_ (where x is an unknown acetyl position); * = Reagent.

N-glycosylation involves attaching N-acetylglucosamine (GlcNAc) to asparagine residues, forming a core structure with GlcNAc and mannose units. This structure is further modified with other carbohydrate structures like fucose, galactose, and sialic acid. Sialic acid (N-Acetyl neuraminic acid) is a 9-carbon chain monosaccharide family that derives from Neuraminic acid and is the most common capping in oligosaccharide chains on the cell surface and in serum glycoconjugates. These glycans are crucial for cellular interactions and immune modulation, with receptors like sialic acid immunoglobulin-like lectins (SIGLECs). Sialic acid serves as a biomarker in cardiovascular disease, diabetes, inflammation, and even cancer - as its levels correlate with inflammation in diseases and are upregulated in tumor microenvironments. Chromatography has been recently used to separate and quantify sialic acids in biological samples. (Yeşilyurt et al., 2015; Thomson et al., 2017; Cavdarli et al., 2019). For the sialic acid analysis, the use of fluorescent labels and reverse phase chromatography is often employed to achieve optimal separation, later followed by UV detection, mass spectrometry or fluorescence detection for measurement and quantification. A detailed protocol for sialic acid quantification is provided below, and a typical example of the results is shown in Figure 13.

1. **Sialic acid release.** Release of Sialic Acid and DMB labelling of the samples is achieved using LudgerTag™ DMB Sialic Acid. After the final wash, the pellet containing the biomolecular hard corona complexes is resuspended in 10 μL of PBS to which 25 μL of acetic acid 2 M are added. The sample is vortexed and incubated at 80°C for 2 h in the thermoshaker. After 1 hour, the samples are vortexed and briefly centrifuged to ensure the correct mixing of the solution, they are briefly centrifuged and placed in the thermoshaker. At the end of the incubation time, the samples are centrifuged at 18,000 rcf to separate the NPs and the supernatant is transferred to a new tube. At this step the samples can be labelled or stored at −20°C for 48 h.
2. **DMB labelling.** 20 μL of a solution containing mercaptoethanol, sodium dithionite and DMB are added to 5 μL of each sample, as well as the sialic acid standards provided in the DMB labelling kit. The samples are left in incubation at 50°C for 3 h. Every hour they are briefly vortexed and centrifuged to ensure proper mixing of the solution. At the end of the incubation time, the samples and the standards are quenched with 475 μL and 480 μL of water respectively. The samples are then diluted 1:10 and run on the UHPLC.
3. **LC analysis.** DMB-labelled samples and standards are analysed using UHPLC-FLD. The LudgerSep-uR2 UHPLC column is prepared, with line A containing a solution of acetonitrile:methanol:water (9:7:84) and line B consisting of acetonitrile. The samples are stored at 4°C in the autosampler, while the oven containing the column is set to 30°C. The fluorescence detector parameters are adjusted to an excitation wavelength of 373 nm and an emission wavelength of 448 nm. The UHPLC gradient is established as per the guidelines provided in Table 3.

**TABLE 3:** Sialic acid quantification UHPLC running 15 min method used with the LudgerSep-uR2 column.

Time (min)	Flow (mL/min)	Solvent A %	Solvent B %
0	0.25	100	0
7	0.25	100	0
7.5	0.25	10	90
8	0.25	10	90
8.5	0.25	100	0
15	0.25	100	0

## 5 Summary and conclusions

This article provides a detailed exploration of protocols for the isolation and characterization of NP-PC complexes. It offers a guide for researchers investigating the interactions between engineered NPs and biological environments through understanding the formation and composition of the PC. a summary table of the techniques covered in this article is provided in Table 4. Accounting the comprehensive methodologies for isolating NP-HC complexes, alongside protocols for the physico-chemical and biochemical characterization of NPs, including glycan profiling and sialic acid quantification protocols.

**TABLE 4:** Summary of the protocols for isolation and characterization of NPs in complex biological media.

Technique	Pros	Cons
Centrifugation	It efficiently separates NP-HC complexes by size and density, using multiple washing steps to remove loosely bound proteins while retaining tightly bound ones. It is versatile, simple, and cost-effective for isolating NPs from diverse biological samples. It offers rapid results and can be scaled for both small-scale experiments and large-scale production, making it adaptable to different research demands	It can lead to particle aggregation or protein sedimentation, especially under excessive centrifugal forces, large centrifugation times or inappropriate conditions. Furthermore, it may struggle with limited resolution when separating NPs from biological samples containing biomolecules of similar sizes and densities, potentially resulting in contamination or incomplete isolation
MACS® Column	Suitable for superparamagnetic NPs. Retention within the column is achieved through external magnetic forces. Subsequent washing steps remove unbound proteins and surrounding solvent constituents. Elution of the NP occurs upon removal of the magnetic field	The protocol necessitates an additional centrifugation step for concentrating purposes, owing to the substantial volume of diluted sample collected during the elution step. It is incompatible with non-magnetized NPs
MagBed	Utilizes ferromagnetic beads to isolate superparamagnetic NPs from complex mixtures. The process is conducted within a microcentrifuge tube, minimizing sample loss and allowing for easy purification steps	It is incompatible with non-magnetized NPs
Size Exclusion Chromatography (SEC)	Fractionates molecules based on size using a stationary phase of pore beads. Larger molecules elute faster, while smaller molecules take longer, allowing for the separation of NPs from proteins and other biomolecules. Suitable for a wide range of molecular weights, and can be particularly effective for small NPs and low-density NPs that would require long centrifugation times and high centrifugation speed. SEC with FPLC instrument is generally connected to a UV detector that measures protein elution and NPs’ scatter and a sample collector allows fraction isolations and further studies. SEC by gravity is cost effective and can be carried out without the need of an FPLC.	A general limitation is the need to find the ideal sample matrix that will not interfere with the NPs and that will allow an ideal separation of the nanomaterials from the biomolecules from the exposing media. FPLC-SEC may not be available in every lab and the NPs have the potential of interfering with the detector. A cleaning protocol must be carefully designed to clean up possible NPs’ deposition and to avoid possible damage of the instrument. SEC by gravity is less cost effective as the separation is manual
Dynamic Light Scattering (DLS)	Time effective Quick and well-established non-invasive method to characterize the hydrodynamic size distribution. Polydispersity values indicate the stability of the samples. <0.1: monodisperse, between 0.1 and 0.4: moderately monodisperse. >0.4: polysdisperse. The sample can be collected after the measurement	The technique is not always applicable to measure NPs in complex media, especially when measuring NPs in biological fluid that contains biomolecules close to the NPs’ size. The data interpretation is not trivial for complex mixtures
Differential Centrifugal Sedimentation (DCS)	High-resolution technique to determine the size distribution of NPs under centrifugal sedimentation. The measurements are highly reproducible as an internal standard with known size and density is run before each sample for calibration. It allows to resolve single NPs from dimers, trimers or aggregates. DCS is one of the few techniques that allow reliable *in situ* measurements as the free unbound proteins will sediment at a significant longer time from the NPs, providing no background. By applying a core-shell model, it is possible to quantify the biomolecular corona thickness in solution. The disc material is compatible with several solutions, and it is possible to use gradients with different composition, even organic solutions. The centrifugation speed can be tailored to allow the sedimentation in a relative short period of time (typically 5 min–30 min) for particles of different sizes and density	Setting up the experiments require the loading of the disc, the gradient stabilization and calibration after each measurement therefore it is not the most time effective instrument. The instrument maintenance is really important as sucrose deposit left over in the disc can lead to disc imbalance and failure. In order to correlate the sedimentation time with size, it is necessary to know the NPs’ density and this value is not always available for composite or core-shell materials. However, it is possible to refer to apparent size shift and measure relative shift before and after corona formation. Measurements can be long for NPs with low density (e.g., polystyrene). The use of a low density disc is recommended to characterize low density material ∼1 and with a minimum particle size of ∼40 nm
Transmission Electron Microscopy (TEM)	Characterization of NPs’ size, shape, and even the protein corona layer. It provides number-based particle size distribution. The size values are closer to the core size of the particles. Cryo-TEM allows the visualization of PC in its native state	TEM requires ultra-clean samples for high-quality imaging, as artifacts from preparation can degrade image quality. Its resolution limits hinder the accurate characterization of protein corona composition. Its static snapshots impede capturing dynamic corona formation. Quantifying parameters such as amount of protein adsorbed is challenging with TEM alone, requiring complementary techniques for thorough analysis
Agarose Gel Electrophoresis (AGE)	Cost-effective method for separating NP-conjugates by size, shape, and charge via an electric current through a gel. Useful for purifying NPs, characterizing particle size, colloidal stability, bioconjugation, and assessing stability	It separates biomolecules based on their size, but it may not provide sufficient resolution to separate NPs from similar-sized biomolecules in complex media. NPs extraction from the gel after the separation can lead to sample loss. AGE does not identify specific proteins in the corona and is unsuitable for quantitative analysis
SDS-PAGE	Qualitative and semi-quantitative information about sample protein composition and abundance, both in fluids and in the NP corona. It is frequently used in proteomic MS analysis as well. It allows a good separation between corona proteins and NPs, that are generally too big to enter into the separating gel	It lacks resolution for distinguishing closely related proteins in the corona, especially with numerous proteins of similar molecular weights. Additionally, the molecular weight does not offer information about the exact identity of the proteins present in the corona
Mass Spectrometry (MS)	Proteomics tool for qualitative and quantitative analysis of complex protein samples through peptide fragment analysis. MS separates ions based on mass-charge ratio (m/z), displaying mass spectra	In MS analysis, proper sample preparation is critical to avoid contamination and loss of information. In the case of complex biological samples, abundant proteins can overshadow signals from less abundant ones, posing a challenge for detecting low-abundance proteins
Glyco-profiling	N-glycan analysis in the corona, important for physiological and immune processes. Most used techniques include hydrophilic interaction chromatography (HILIC) and mass spectrometry. Key for understanding NPs’ interactions in biomedical applications	Involves sequential and delicate preparation steps, which can sometimes lead to sample loss. Labelled samples need to be analyzed as soon as possible to prevent quenching and achieve good resolution. The process demands well-trained researchers to conduct the instrument calibration, column and solvent selection, separation optimization, and chromatogram interpretation
Sialic Acid Quantification	Analysis of sialic acids, a key monosaccharide in cellular interactions and immune modulation. Chromatography, usually paired with fluorescent labeling techniques, is used for separation and quantification. Relevant for its role as a biomarker in various diseases	Labelled samples need to be analyzed as soon as possible to prevent quenching and achieve good resolution. The process demands well-trained researchers to conduct the instrument calibration, column and solvent selection, separation optimization, and chromatogram interpretation

The biomolecular corona is becoming a key tool to predict and enhance the biocompatibility and functionality of engineered NPs. Future challenges remain the need to promote tested protocols and methods that avoid artefacts and common mistakes, such as biomolecular carryover in the background, failing to report a non-realistic condition and in reporting the data using standardised framework. (Faria et al., 2018). In this sense, future research should also try to understand better the dynamic protein exchanges on the surface of the NP, not only during the formation of the PC but on the biochemical changes occurring at longer timescales and also under flow condition. Controlling this item would impact the cellular uptake and signaling pathways, allowing to develop NPs designed to exploit specific corona effects. Artificial intelligence (AI) will play a key role in all these advancements thanks to its capacity for handling complex and large datasets. Furthermore, AI and high performance computing (HPC) can significantly enhance the design and optimization of NPs by modeling and simulating their interactions in biological systems, as well as predicting the formation and dynamics of the biomolecular corona. We believe that the current set of protocols for isolation and characterization of NPs and their corona in complex biological media will be helpful to achieve all future goals and pave the way for safer and more effective nanomaterials.

## Data Availability

The original contributions presented in the study are included in the article/Supplementary material, further inquiries can be directed to the corresponding authors.
